# Genome-wide analysis of AP2/ERF family in *Akebia trifoliata* and characterization of an *AtrERF001* gene regulate fruit ripening

**DOI:** 10.3389/fpls.2025.1607254

**Published:** 2025-08-26

**Authors:** Juan Niu, Ying Li, Zhimin Sun, Ying Lin, Chao Zhang, Bo Yang, Shuang Tian, Mingbao Luan

**Affiliations:** ^1^ School of Biological and Environmental Engineering, Jingdezhen University, Jingdezhen, Jiangxi, China; ^2^ Institute of Bast Fiber Crops, Chinese Academy of Agricultural Sciences, The Southern Characteristic Crop Genetic Species Innovation Team, Changsha, China; ^3^ State Key Laboratory Breeding Base of Dao-di Herbs, National Resource Center for Chinese Materia Medica, China Academy of Chinese Medical Sciences, Beijing, China; ^4^ School of Life Sciences, Shangrao Normal University, Shangrao, Jiangxi, China; ^5^ National Nanfan Research Institute, Chinese Academy of Agricultural Sciences, Sanya, China

**Keywords:** *Akebia trifoliata*, AP2/ERF family, expression profile, fruit ripening, transcriptional regulation

## Abstract

**Introduction:**

Ethylene response factors (ERF) were important for plant growth, hormone signaling, fruit ripening and stress response. Despite the wide identification of ERF family members in various species, limited information is available regarding this family in *Akebia trifoliata*.

**Methods:**

The APETALA2/ethylene response factor genes in *A. trifoliata* were identified and analyzed using bioinformatic approaches and *AtrERF001* was verified to be involved in fruit ripening by experiments.

**Results:**

Therefore, 131 APETALA2/ethylene response factor genes were identified from the *A. trifoliata* genome. Gene structures, motif compositions, tandem duplication events and promoter structure of ERF genes were characterized, providing insights into the molecular basis underlying the discrepant functions of ERF genes within each evolutionary branch. Expression patterns under ethylene and 1-MCP treatments of these *ERF* genes were further analyzed using real-time PCR (RT-qPCR), revealing that 9 key ERF genes are closely associated with fruit ripening. A co-expression regulatory network analysis indicated that *AtrERF001* was one of the hub gene. *AtrERF001* was found to localize in nucleus by subcellular localization analysis. Overexpression of *AtrERF001* in *Akebia trifoliata* and tomato fruit resulted in early fruit ripening. The expression levels of *AtrERF001*, *AtrACO* and *AtrPE* in overexpression fruits were increased by about 30-fold, 5-fold and 1-fold, respectively, compared with the control, whereas silencing of *AtrERF001* in *A. trifoliata* by virus induced gene silencing showed opposite trends. Moreover, RT-qPCR experiments showed that the expression of *AtrERF001*, *AtrACO* and *:AtrPE* in *AtrERF001* overexpression tomato fruits at red-ripe stage were significantly increased compared with the control fruits, indicating that *AtrERF001* may play an important role in regulating fruit ripening.

**Discussion:**

Our results provide new insights into the underlying regulatory mechanisms of the AP2/ERF family during fruit ripening.

## Introduction

Transcription factors (TFs) are a class of specific proteins that typically contain one or more specific DNA-binding structural domains. The expression of a target gene is mediated by a specific cis-DNA sequence in the promoter of the target gene (Xu et al., 2024). TFs are essential for gene regulation during plant development and maturation. Ethylene response factor (ERF), one of the largest plant-specific TF families, is a member of the APETALA2/ethylene response factor (AP2/ERF) superfamily and functions as an important downstream element of the ethylene signaling pathway (Liu et al., 2015). The AP2/ERF family can be divided into four subfamilies, ERF, dehydration-responsive element binding (DREB), AP2, and Aintegumenta (ANT), based on the number of AP2 conserved structural domains (Toshitsugu et al., 2006; Dietz et al., 2010). These subfamilies display various structural traits, such as the 14^th^ and 19^th^ amino acids in the AP2 structural domain of the DREB subfamily members are valine (V) and glutamate (E), respectively, while in the ERF proteins are alanine (A) and aspartate (D). The ability of these proteins to selectively bind to the core motif of the DRE (A/GCCGAC) or GCC boxes (AGCCGCC) is important for the transcriptional control of downstream target genes (Wang et al., 2015; Han et al., 2016; Kuang et al., 2017; Wang et al., 2019b).

*ERF* genes have been implicated in various processes in recent years, including plant development and growth, hormone signaling, fruit ripening (Xie et al., 2019; Xing et al., 2021; Hu et al., 2022; Zhang et al., 2022), and stress response (Yao et al., 2017; Zhu et al., 2018; Bajpai et al., 2021). For example, the involvement of *DkERFs* in persimmon fruit ripening is accomplished by regulating ethylene biosynthesis-related genes 1-aminocyclopropane-1-carboxylate synthase, ACC oxidase (ACO), and cell wall-modifying genes-pectate lyase (*PL*), β-galactosidase (BGAL), xyloglucan endo-transglucosylase/hydrolase (*XTH*), and pectinesterase (*PE*) (He et al., 2020). *PpERF/ABR1* regulates fruit softening in peach (*Prunus persica*) fruit during storage by activating downstream *PpPG* expression. *ERFs* may play a role in modulating cellulose production and lignin buildup through the transcriptional control of cell wall-related genes. The related to abscisic acid-insensitive 3/viviparous1 (RAV) family proteins primarily regulate responses to diverse biotic and abiotic stressors (Wang S. et al., 2020; Liu et al., 2021).

Ripening of fleshy fruits involves significant changes in morphological, physiological, and biochemical changes during fruit growth and ripening, such as pigment accumulation, texture, flavor, softening, production of volatile compounds, and nutritional value (Chen T. et al., 2020). This process is important to maintain the quality of the fruit. However, overripening may lead to a reduction in disease resistance and quality. Therefore, understanding the regulatory systems and molecular mechanisms involved in fruit ripening is crucial for creating plans to enhance the nutritional value, sensory quality, and shelf life.

*Akebia trifoliata* (Thunb.) Koidz., which has a high yield, wide adaptability, high seed oil content, ease of management, and high economic, nutritional, and medicinal values, can be used as a medicinal food plant for biodiesel production, vine oil crops, and other applications (Luo et al., 2013; Yan et al., 2014; Wang et al., 2019a; Niu et al., 2021). With the increasing demand for *A. trifoliata* fruit quality, understanding the underlying regulatory mechanisms of fruit ripening has become important. It is hypothesized that ethylene-related genes also play key roles in the maturation of climacteric *A. trifoliata* fruit (Cao et al., 2003; Niu et al., 2020). However, in-depth identification and analysis of the *AP2/ERF* family in *A. trifoliata* has not been conducted.

In this study, a genome-wide analysis was conducted to identify the ERF family according to the *A. trifoliata* genome, resulting in the identification of 131 AP2/ERF genes. These ERF genes were systematically characterized, including phylogenetic relationships, gene structures, conserved motifs, chromosome distribution, gene duplication events, promoter cis-acting elements, their KEGG and GO annotation, as well as their expression profiles and ERFs accumulation during fruit ripening of *A. trifoliata*. Furthermore, a novel ERF involved in fruit ripening was identified and characterized. Functional assays of *AtrERF001 in vitro* and vivo indicated that *AtrERF001* was involved in fruit ripening by directly upregulating the genes of *AtrPE* and *AtrACO*. Our results will help to illustrate the general functions of the ERF family in *A. trifoliata* and provide helpful gene resources for breeding and postharvest preservation.

## Materials and methods

### Identification of AtrAP2/ERF family genes in *A. trifoliata*

To retrieve the AP2/ERF gene sequences, the *A. trifoliata* genome and protein sequences were retrieved under accession numbers PRJNA750300, SAMN20447974. The assembled chromosome-level genome of *A. trifoliata* was 726.85 Mb, consisting of a scaffold N50 of 42.49 Mb and a contig N50 of 2.29 Mb, with a repeat rate of 42% and 39,443 protein-coding genes annotated (Niu et al., 2024). The candidate AP2/ERF proteins were searched from the *A. trifoliata* genomic database by using the Hidden Markov Model (HMM) mapping (PF00847). The AP2/ERF superfamily genes and their corresponding protein sequences of Arabidopsis and rice were retrieved from PlantTFDB (https://planttfdb.gao-lab.org/) and HMMER3.0. (https://www.ebi.ac.uk/Tools/hmmer/home), respectively. The ERF protein sequences of Arabidopsis and rice were 128 and 139, and a BLAST search of the genome of *A. trifoliata* was performed. The obtained sequences were used to confirm the presence of the AP2 structural domain using Pfam, the National Center for Biotechnology Information, and the Conserved Structure Database. Using the SwissProt database, sequences with high E-values were selected for comparison. Finally, as a quality check, the sequences were analyzed through the Simple Modular Architecture Research Tool (SMART) (https://smart.embl.de/smart/change_mode.cgi) to confirm the presence of the AP2 domain. The physical and chemical characteristics, sequence length, molecular weight (MWs), theoretical isoelectric point (pI), and subcellular localization of *AtrAP2/ERF* were predicted using the online analysis tools ProtParam and pLoc-mPlant, respectively.

### Classification, phylogenetic relationships and motif analysis of AtrAP2/ERF proteins

MEGA-X software aligned the AP2 domains of AtrAP2/ERF proteins based on conserved domain sequences. The Jalview program was used to manually correct the predicted amino acid sequences of the AP2 motifs. A maximum likelihood phylogenetic tree was created using MEGA7.0 and the online tool iTOL. *AtERF* and *OsERF* classification systems were used to classify identified *AtrAP2/ERF* genes into several groups. The NCBI CDD and MEME program was used to obtain the AP2/ERF conserved domains and motifs, respectively. The biological data toolkit TBtools was used to visualize the exon-intron arrangements, protein structures, motifs, and chromosomal locations of the *AtrAP2/ERF* genes (Chen C. et al., 2020).

### Chromosome distribution, gene duplication, and collinearity analysis of *AtrAP2/ERF*

The chromosomal locations of the AP2/ERF gene family members were extracted from the gff3 file of the *A. trifoliata* genome annotation, and a map of the chromosomal gene distribution was constructed with Circos software (Feng et al., 2022). Gene duplication and collinearity between *A. trifoliata* and other species (*A. thaliana*, *Oryza sativa*, and *Aquilegia vulgaris*) were examined by using MCscanX (Wang et al., 2012). The synonymous (Ks) and non-synonymous (Ka) substitutions in each duplicated *AtrAP2/ERFs* were determined by the KaKs calculator (version 2.0) (Wang et al., 2010).

### Cis-element of *AtrAP2/ERF* gene family and functional enrichment analysis of *AtrAP2/ERF* target genes

The 2.0-kb sequence upstream of the promoter region was analyzed in the PlantCare database to obtain the cis-element of the *AtrAP2/ERF* gene. TBtools was used to examine the location and numbers of the cis-element classified into three main categories: stress-responsive, developmental-responsive, and phytohormone-responsive. The Gene Ontology (GO) and Kyoto Encyclopedia of Genes and Genomes (KEGG) databases was used to identify the potential *AtrAP2/ERF* target genes.

### Expression profiles and interaction networks of *AtrAP2/ERF* genes

The *A. trifoliata* transcriptome data were used to examine the expression patterns of *AtrAP2/ERF* genes in different tissues and stages (**Supplementary Table S1**). For tissue-specific RNA-Seq analysis, nine tissues including different fruit cracking period (non-cracked fruits, PS; initially cracked fruits, PM; totally cracked fruits, PL) (Niu et al., 2020), flesh stages (hard flesh: UR; pericarp begins to soften; however, the flesh is hard: HR; soft flesh: FR) (Niu et al., 2021), and seed development (August 20: T; September 4: U; September 19: I) (Zhong et al., 2022) were considered and the raw data were retrieved under the accession number PRJNA524995, PRJNA750300 and PRJNA79843. All the transcriptome sequencing data were obtained from the HiSeq 2500 sequencing system. The raw data were filtered to trim low-quality reads and discard transcripts less than 200 bp in length. The remaining clean reads were mapped to the reference *A. trifoliata* genome using HISAT2 or String Tie, to obtain unigenes. P-Unigene expression levels were calculated as fragments per kilobase of exon model per million mapped fragments (FPKM) using the Cufflinks software package, and Hochberg’s approach to adjust the p value. The differential expression of *AtrAP2/ERF* genes was visualized by creating heat maps using TBtools. Using the STRING 11.5 database (http://string-db.org), cluster analysis and functional network analysis were performed on the orthologous gene pairs between *AtrERF* and *AtERF*, and the interaction network was displayed (Damian et al., 2017). Spearman’s correlation was used to predict the key genes for *AtrAP2/ERF* based on the degree of association between the genes.

### Quantitative (real-time) PCR analysis of genes under ethylene and 1-methylcyclopropene treatment of *A. trifoliata* fruits

Plants with consistent growth size, tree age, and fruit development were used for the ethylene and 1-MCP treatments, and a minimum of 45 fruits per plant were chosen as the experimental unit. These plants were used 20 days before fruit harvesting. Ethylene (300 mg/L), 1-MCP (10 mg/L), abscisic acid (ABA) (0.5 mMol/L) and its inhibitor fluridone (0.5 mMol/L) were sprayed on September 12, 2021, with water as the control. On October 2, three replicates of the samples were collected for RT-qPCR analysis.

Total RNA was extracted and reverse transcribed into cDNA using Plant RNA Extraction Kit (Accurate Biotechnology Co., Ltd., Hunan, China) and Evo M-MLV RT, respectively, followed by RT-qPCR analysis using the SYBR^®^qRT-PCR Kit (Accurate Biotechnology Co., Ltd., Hunan, China) on a multicolor Real-Time PCR Detection System (Bio-Rad Laboratories, Berkeley, CA, USA). In this study, the *EF-1α* gene was used as the internal control gene, which was detected by the *de novo* transcriptome sequencing of *A. trifoliata* (Yang et al., 2016). The gene primer sequences are listed in **Supplementary Table S2**. The 2^-ΔΔCt^ formula was used to calculate the gene expression and the histograms were made using GraphPad Prism 8 software. The gene network correlation analysis of key *AtrERF* genes was displayed by Gephi 0.9.5 (Kauffman et al., 2014).

### Subcellular localization

The full-length open reading frame sequence (ORF) of *AtrERF001* (missing stop codon) containing the BamHI locus was inserted into the PBI21-CaMV35S-GFP vector to generate the PBI21-*AtrERF001*-GFP recombinant plasmid. The expression vector PBI21-*AtrERF001*-GFP after transformation of *Agrobacterium tumefaciens* receptor cells GV3101 was suspended to OD_600_ = 0.8-1.0 after expanded culture with MES buffer (10 mM MgCl_2_, 10 mM MES, pH 5.6; 150 μM acetosyringone), and after infesting the leaves of *Nicotiana benthamiana* for 36–48 h, then cut the tobacco leaf near the injection hole with scissors and place it on a slide. The fluorescent protein in tobacco cells was observed by laser confocal electron microscopy to determine the subcellular localization of the *AtrERF001* gene.

### Yeast one-hybrid assays

The ORF sequence of *AtrERF001* (missing stop codon) and the prey vector was constructed by ligating it to the pGADT7 vector using the EcoRI and BamHI restriction sites. The bait vector was constructed by cloning the promoter regions of *AtrACO1* (500 bp) and *AtrPE* (708 bp) containing the *AtrERF001* binding pattern (GCCGAC) into the pAbAi vector using SmaI and SalI restriction endonucleases. The recombinant bait-pAbAi plasmid was then digested using the BstBI endonuclease and transformed into the Y1H yeast strain. Y1HGold [bait/AbAi] positive strains were verified using colony PCR. The inhibitory concentrations of aureobasidin A (AbA) were evaluated by distributing gradient concentrations on SD/-Ura/AbA plates. The recombinant pGADT7-*AtrERF001* plasmid was then transformed into the Y1HGold strain made from the bait recombinant vector and cultured on SD/-Leu/AbA plates. Protein-DNA interactions were determined based on the ability of transformed yeast cells to grow on SD/-Leu/AbA medium. The interaction of pGADT7–53 and p53-AbAi, pGADT7 and AbAi, were used as positive and negative controls, respectively. All transformations and screenings were performed at least three times. Primers used for this assay are listed in **Supplementary Table S3**.

### Electrophoretic mobility shift assay (EMSA)

The full-length coding sequence of *AtrERF001* was cloned and inserted into the pET-32A vector and transformed into *Escherichia coli* strain BL21 (DE3) (Novagen, Denmark). Protein expression was induced using 0.2 mM isopropyl b-D-1-thiogalactopyranoside (IPTG) at 16°C for 20 h. The recombinant protein was purified with a Capturem His-Tagged Purification Miniprep Kit (Clontech, CA). The triple tandem copies of the coupled element 1 (*AtrACO1*) motif (FP1:AATTTGAGTCGGCATTTTTT, RP1:AAAAAATGCCGACTCAAATT) and *AtrPE* motif (FP2:TTCCAAGCCGACATGGCCGA, RP2: TCGGCCATGTCGGCTTGGAA) were labeled with biotin at the 5′ terminus (BioRun, Wuhan, China) and used as a probe, and the unlabeled same sequences was used for the competition assay. The specificity of binding was examined by competition with the unlabeled probes (10×, 50×). Electrophoretic mobility shift assay (EMSA) was performed using a Light Shift Chemiluminescent EMSA kit (Thermo Scientific, MA) according to the manufacturer’s instructions.

### Overexpression and VIGS vector construction and genetic transformation

After amplifying the *AtrERF001* coding region with cloning primers (**Supplementary Table S4**), the PCR product was ligated into a cloning kit (TransGen Biotech, China). The ORF sequence of *AtrERF001* was ligated into pBI121 and pTRV2 vectors that had been digested with BamHI and transformed into *Agrobacterium tumefaciens* receptor cell EHA105. The injection of *AtrERF001* on *A. trifoliata* fruits was performed as previously described for cherry fruits (Qi et al., 2017). Strains carrying overexpressed (PBI121-*AtrERF001*) and repressed expression vectors (pTRV2-*AtrERF001*) were cultured and expanded on LB medium to an OD_600_ of 1.0-1.5 at 28°C. Then bacterial solution was resuspended in 2-Morpholinoethanesulphonic acid (MES) infiltration buffer with a final OD_600_ of 0.8-1.0. Suspensions containing pTRV1+pTRV2, pTRV1+pTRV2-*AtrERF001*, PBI121 and PBI121-*AtrERF001* were infiltrated into *A. trifoliata* fruits at 130 days (September 20, 2022) after flowering using a syringe, respectively. The injected fruits were bagged to increase the humidity of the surrounding air. Three replications, each replicate injected with 9 fruits were used for the experiment from 8-year-old fruit trees in the same area.

### Extraction and determination of cell wall components of *A. trifoliata* pericarp

Determination of pectin content was described as previous studies (He et al., 2020). One gram of powder sample was washed twice with 95% ethanol (v/v) in a boiling water bath for 30 min. After centrifugation, the precipitate was resuspended in 30 ml of distilled water and heated at 50°C for 30 min. The supernatant after centrifugation was used to determine the water-soluble pectin (WSP) content. The remaining precipitate was dissolved in 25 ml H_2_SO_4_ (0.5 M) and boiled for one hour in a boiling water bath. Acid-soluble pectin (ASP) content was measured in the supernatant after centrifugation. WSP and ASP were determined using the colorimetry of the carbazole–sulfuric acid technique. Hemicellulose and cellulose content were measured as described in a previous study (Sun et al., 2020). After centrifugation, cellulose and hemicellulose were separated with dilute hydrochloric acid (HCl). Firstly, the samples were dissolved in 20 mL of 2N HCl, and hemicelluloses were measured in the supernatant. Seventy-two percent H_2_SO_4_ was used to dissolve the remaining residue and incubated for 1 h. After filtration, the cellulose content of the supernatant was measured. Finally, the cellulose and hemicellulose contents were determined using the anthranilic sulfuric acid method.

### Tomato regeneration and transformation

Mid-sections of cotyledons (7–8 days after germination) of wild-type tomato plants (*Lycopersicon esculentum* Mill. cv. Micro-Tom) were used as material following previously described methods (Liu et al., 2020). After a total of three days of incubation, transformed leaf healing tissues were transferred to regeneration medium by culturing on Murashig and Skoog (MS) medium (MS+100 mg/l kanamycin+1.0 mg/l ZT+1.0 mg/l IAA+300 mg/l Timentin). After rooting, the regenerated plantlets were transferred to soil (**Supplementary Figure S1**). RNA was isolated from the leaves of wild-type and transgenic plants for PCR validation, and six major transgenic lines were identified, and T-26 was confirmed to carry the inserted *AtrERF001* transgene. The first-strand cDNA of different developmental stages of fruits was synthesized and used in RT-qPCR analysis.

### Statistical analyses

Three biological replicates were collected for each sample and the data in the figures were plotted with mean ± standard deviation. Statistical analysis was carried out by ANOVA SPSS 21.0 software (Chicago, IL, USA), and significant differences at p < 0.05 were determined using Duncan’s multiple range tests. The asterisks represented extremely remarkable differences (**: p< 0.01; ***: p< 0.001) between the different samples.

## Results

### Identification and classification of *AtrAP2/ERF* genes

Based on HMM, CDD and Pfam data, 131 *AP2/ERF* genes were identified in *A. trifoliata* according to the number of AP2 domains. These genes were renamed AtrAP201 to AtrAP221, AtrERF001 to AtrERF108, and RAV1 to RAV2 according to their corresponding positions on the chromosome. The further characterization of the protein length, molecular weight and isoelectric point (PI) reported that the AtrAP2/ERF proteins included 121–709 amino acids, with an MW of 13.58–79.65 kDa and a projected PI of 4.5–10.7. The *AtrAP2/ERF* TFs are predicted to be localized in the nucleus (**Supplementary Figure S2**; **Supplementary Table S5**). Multiple sequence alignments showed that the conserved sites-Gly (G)-4, Arg (R)-6, Glu (E)-16, Trp (W)-28, Leu (L)-29, G-30, and Ala-38 are present in most of the 131 AP2/ERF family proteins (**Supplementary Figure S3**). The AP2/ERF family can be divided into two main groups, group A (I, II, III, IV) and B (V, VI, VII, VIII, IX, X, XI-L) based on the phylogenetic tree created according to *A. thaliana* and *O. sativa* and ERF groups (Toshitsugu et al., 2006). Groups A and B contained 40 and 91 *AtERFs*, respectively, and each subfamily tended to have one or more homologs or orthologs in *A. thaliana* and *O. sativa*, respectively. The ERF groups for *A. trifoliata* were highly consistent with the phylogenetic tree, indicating its authenticity. However, no *AtrERF* genes belonging to the Vb-L subgroup were observed (**Supplementary Figure S4**).

### Analysis of *AtrAP2/ERF* family gene structure and component composition

The gene structure of *AtrERF* family members was investigated by comparison with genomic DNA sequence to learn more about their exon-intron and evolutionary structures. A total of 102 genes contained coding region sequences (77.9%), 29 of which were interrupted by introns. While none of the genes in Group I or III had introns, all genes in Group VII have two introns. A total of 20 conserved motifs were identified in the *AtrAP2/ERF* family using the MEME online software. All 131 proteins, except ERF030 and ERF089, possessed motifs 1 and 2. Most AtrAP2/ERF proteins in the same subgroup share a common motif, such as motifs 1, 2, 3, 4, and 5 (groups VI–L), motif 20 (Group III), and motif 19 (Group IX) on the phylogenetic tree, indicating that these ERF proteins have a similar evolutionary relationship (**Supplementary Figure S5**).

### *AtrAP2/ERF* gene family synteny analysis

**Figure 1:**
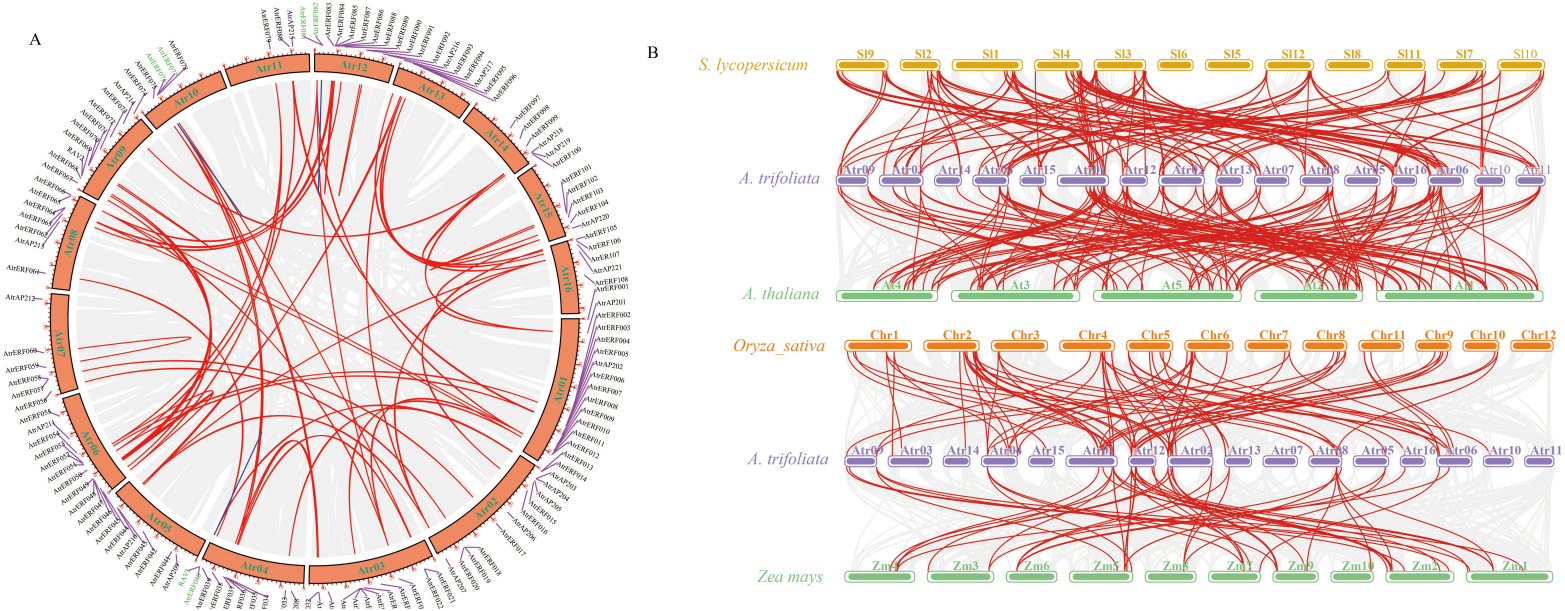
The gene duplication analysis of the *A. trifoliata* AP2/ERF gene family. **(A)** Chromosome distribution and duplication analyses of the *AtrAP2/ERF* genes of *A. trifoliata*. Gray lines indicate all synteny blocks in the *A. trifoliata* genome, tandemly duplicated *AP2/ERF* gene pairs are connected with red lines, and blue lines indicate segmental duplicated gene pairs. **(B)** Analysis of *AP2/ERF* gene duplication and synteny in *A. trifoliata* and four representative plant species. Gray lines in the background indicate the collinear blocks within *A. trifoliata* and other plant genomes, while the red lines highlight the syntenic *AP2/ERF* gene pairs. The species included: *A. trifoliata*, *Arabidopsis thaliana*, *Solanum lycopersicum*, *Oryza sativa*, and *Zea mays*.

Gene expansion and the creation of novel functionalities greatly benefit gene replication. A total of 58 pairs of gene repeats were identified in *A. trifoliata*, which were divided into tandem repeats and segmental duplication. There were only 3 pairs of tandem repeats (TD, 5.2%), while the remaining 55 pairs were classified as segmental duplicated pairs (SD, 94.8%) (**Figure 1A**; **Supplementary Table S6**), suggesting that SD made a valuable contribution in the gene family evolution. Synonymous substitution rates (Ka/Ks) of gene pairs are useful tools for understanding evolutionary dynamics after the occurrence of gene duplication. Except for *AtrERF047/049* (Ka/Ks = 1.66, > 1 correlates with positive selection), the Ka/Ks ratio for tandem and segmental duplicated gene pairs in *A. trifoliata* was less than 1 (Ka/Ks < 1 indicates purifying/negative selection) (**Supplementary Table S7**), suggesting negative selection pressure. Comparative syntenic maps of the representative dicot and monocot plant species *A. trifoliata*, *A. thaliana*, *Solanum lycopersicum*, *O. sativa* and *Zea mays* indicated that all four species shared 41 collinear gene pairs (**Supplementary Table S8**; **Figure 1B**). *A. trifoliata* shared 91 orthologous gene pairs with monocotyledons (54/37) and 154 with dicotyledons (88/66). Furthermore, 95% (39/41) of the identified gene pairs between *A. trifoliata* and dicotyledons differed from those shared by *A. trifoliata* and monocotyledons, indicating that these ERF orthologous genes evolved after the divergence of dicots from monocots plants.

### Analysis of the Cis-acting elements

**Figure 2:**
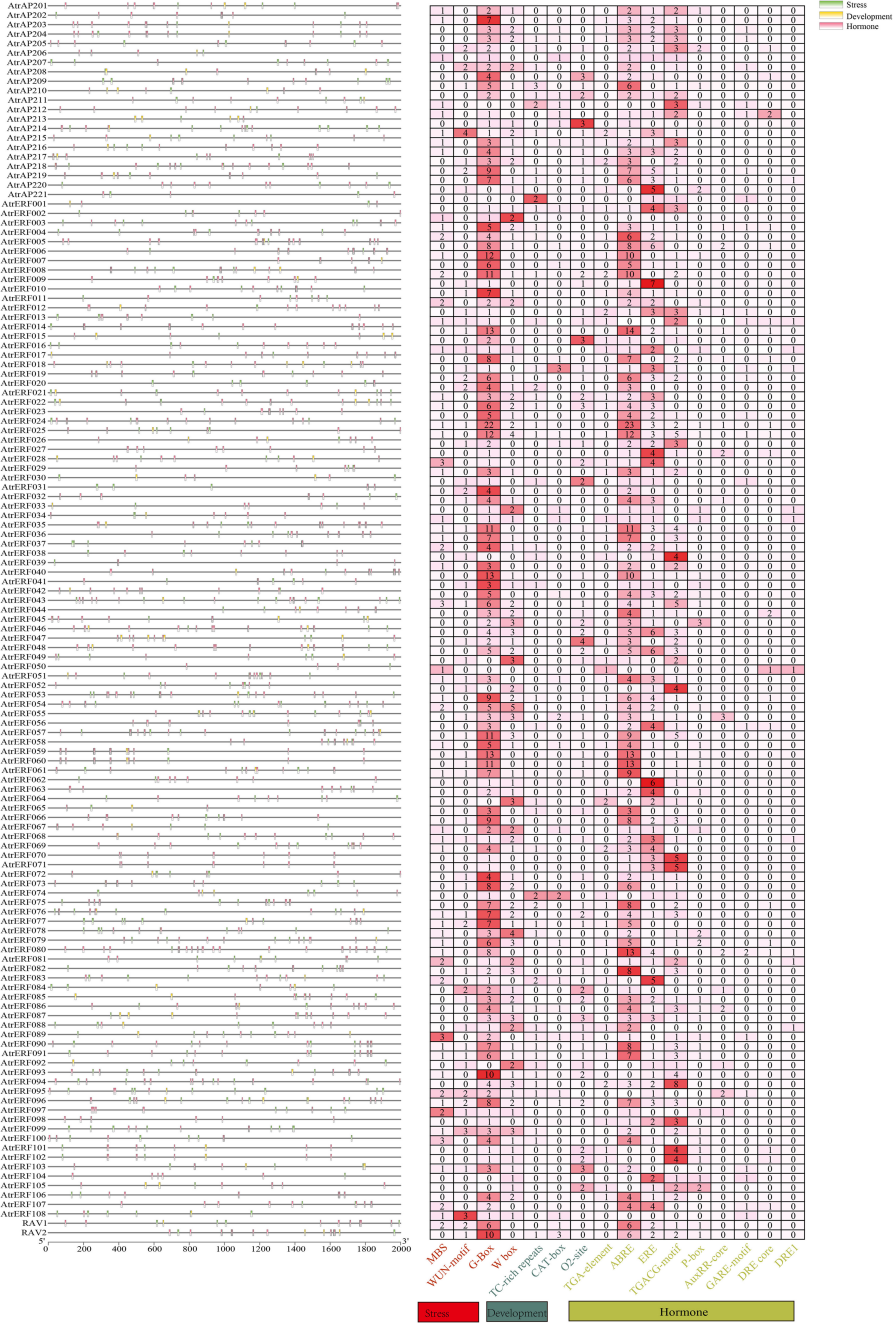
Analysis of cis-elements in the promoter region of the *AtrAP2/ERF* genes. The distribution of upstream promoter regions of the three cis-acting elements is shown on the left, and the heat map of cis-elements for phytohormone response, stress response, and plant growth is shown on the right.

The cis-regulatory elements in promoter sequences were analyzed using PlantCARE software, leading to an understanding of the evolutionary and functional diversification of the *AtrAP2/ERF* genes. A sum of 16 cis-acting elements were identified from the promoter region of *AtrAP2/ERF* genes, which was divided into three groups: phytohormone-responsive (including TGA-element, abscisic acid-responsive: ABRE, ethylene-responsive: ERE, TGACG-motif, GARE-motif, DRE core: GCC box, DRE1:dehydration responsive element/C-repeat, P-box, and AuxRR-core), stress-responsive (including MBS, WUN-motif, G-Box, and W-box), and related to plant development (including the CAT-box and O2-site). Moreover, the AP2 domain of ERF can bind to multiple cis-elements present in the promoter of ethylene-responsive genes, such as GCC box and DRE/CRT, and activate or inhibit the expression of these genes to regulate fruit development and ripening. Most of the *AtrAP2/ERF* genes contain light-responsive (G-box), ABRE, and ERE elements, which were the most prevalent cis-acting elements in phytohormone-responsive plants (**Figure 2**, **Supplementary Table S9**), indicating that the *AtrAP2/ERF* genes play a significant role in light, stress, hormones, and plant development, as evidenced by the composition of their cis-acting elements.

### Prediction of potential target genes of *AtrAP2/ERF* and their functional enrichment analysis

The assembled *A. trifoliata* genome contained 28,411 genes with at least one ERE (ATTTTAAA) and one DRE (ACCGAGA/GCCGAC/TACCGACAT) in their putative promoters. Of these, 4499 genes had a CCGAC core motif, and 7836 genes had at least one DRE. Consequently, 4499 genes were selected for additional functional enrichment analyses (**Supplementary Table S10**). A few target genes were enriched in stress response and polysaccharide metabolic pathways in the GO terms (**Supplementary Figure S6A**). Numerous target genes were enriched in environmental adaptability and plant hormone signal transduction according to the KEGG pathway analysis (**Supplementary Figure S6B**). Protein-protein interactions provide intuitive and rapid understanding of the gene function of family genes, and are also important for the regulatory network relationship between family proteins. To predict the molecular interactions between *AtrAP2/ERF* and other proteins, the PPI network of *AtrAP2/ERF* and cell wall related genes was constructed based on Arabidopsis homologous proteins (**Supplementary Figure S7**). It was predicted that cell wall, and ethylene signaling pathway related genes interacted with *AtrAP2/ERF* genes in the networks. For example, *AtrERF043* can interact with *AtrNAC*, *AtrPL4*, *AtrPE3*, *AtrACO*; *AtrERF001* can interact with *AtrPE4*, *AtrPL2*, *AtrCYP707A2* (**Supplementary Figure S7B**), which played roles in fruit ripening.

### Analysis of the expression pattern of *AtrAP2/ERF* gene during fruit ripening

Transcriptome data from several developmental stages were used to study the expression patterns of the *AtrAP2/ERF* genes, with some genes preferentially expressed in the detected tissues. For example, five (23.8%), nine (42.9%), and seven (33.3%) of these were expressed (FPKM value >1) in the pericarp, pulp, and seed, respectively. Among the 131 *AtrAP2/ERF* genes, 11 were expressed in all nine samples evaluated (FPKM >0). Fourteen genes (*AtrERF011/014/019/027/028/031/035/063/074/076/077/093/101/102*; *AtrAP203/04*/13) identified in the seed samples revealed significant transcript abundance during fruit ripening. The highest transcript abundance (FPKM >2 in at least one of the other tissues) was seen in three genes (*AtrERF005/020/048*; *AtrAP203/04*/13) in the initial crack stage (PM), ten genes in the complete cracking stage (PL) (*AtrERF001/009/015/018/034/043/054/068/082/090*; *AtrAP214*), five genes in the HR pulp stage (*AtrERF019/020/027/031/035*), and three genes in the mature pulp stage (FR) (*AtrERF005/079/080*; *AtrAP213*) (**Supplementary Figure S8**, **Supplementary Table S1**). Our study found that 14 *AtrAP2/ERF* genes were significantly upregulated at different developmental stages, and the highest expression levels were observed during the mature period.

### Ethylene and 1-MCP treatment altered *AtrAP2/ERF* gene expression profiles in fruit

**Figure 3:**
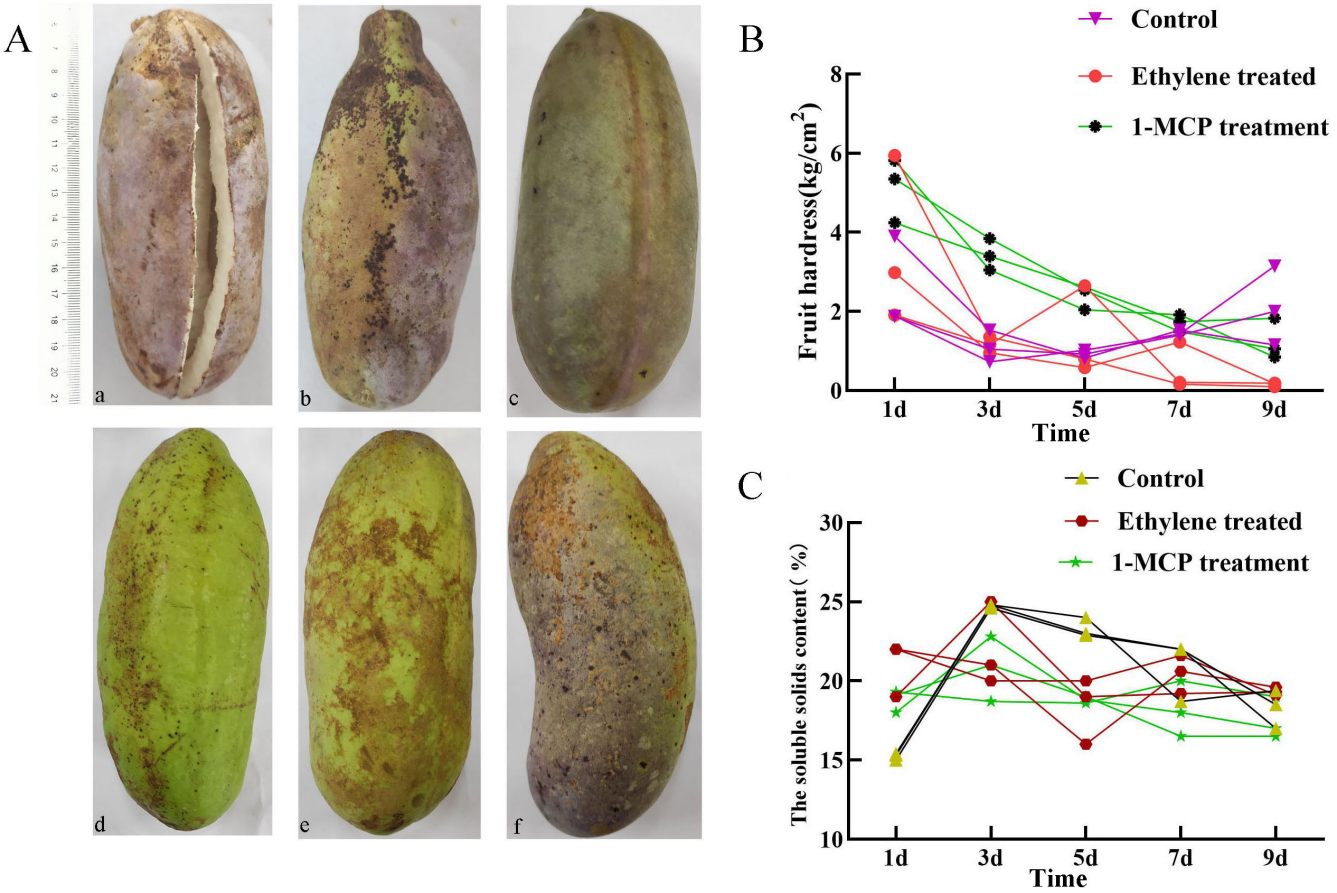
Phenotypic observation and fruit measurement of fruit traits of *A. trifoliata* after different treatments. **(A)** Phenotypic observation of *A. trifoliata* fruit after different treatments. (a) ethylene, (b, e) control fruit, (c) 1-methylcyclopropene (1-MCP) treatment, (d) abscisic acid and (f) inhibitor fludioxonil. **(B)** Fruit hardness determination after different treatments. **(C)** Determination of fruit soluble solids content after different treatments.

Analysis of cis-acting elements showed that abscisic and ethylene response elements were the most prevalent cis-acting elements in *AtrAP2/ERFs*. To verify the effect of different hormone treatments on the ripening in *A. trifoliata* fruit, ethylene and its inhibitor 1-MCP, abscisic acid and its inhibitor fludioxonil were treated on *A. trifoliata* fruits 20 days before fruit ripening. Ethylene treatment (a) led to early fruit ripening, whereas 1-MCP treatment (c) delayed it. Abscisic acid (d) and its inhibitors (f) had little effect on fruit ripening (**Figure 3A**). The changes of fruit hardness and soluble solids content under different treatments were further analyzed. It was found that the hardness of ethylene-treated fruits was lower than that of the control group, while the soluble solids content increased significantly (**Figures 3B, C**). It indicated that ethylene-based treatment could accelerate fruit ripening and did not affect the edible taste of the fruit, while the results of 1-MCP treatment were just the opposite.

**Figure 4:**
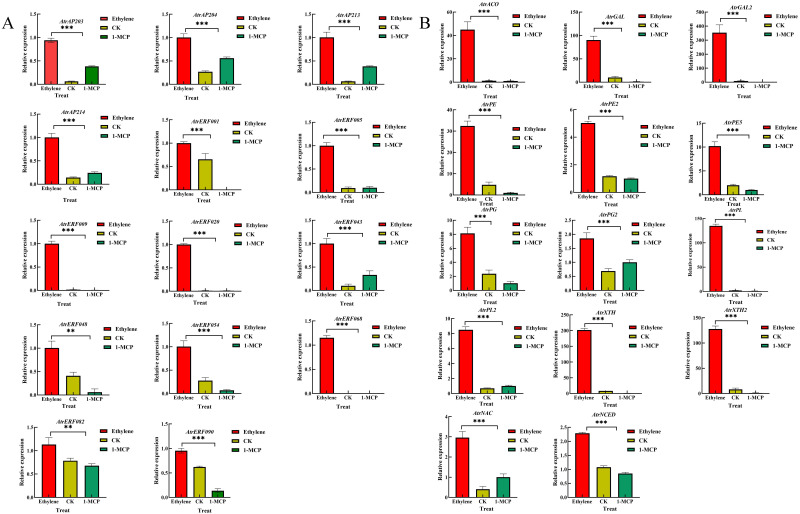
RT-qPCR analysis of selected *AP2/ERF* and cell wall-related genes in *A. trifoliata* pericarp under ethylene and 1-MCP treatment. **(A)** RT-qPCR analysis of selected *AP2/ERF*. **(B)** RT-qPCR analysis of selected cell wall-related genes in *A. trifoliata* pericarp. Error bar represents the standard deviation of the three biological duplicates, **represents p <0.01, ***represents *p <*0.001.

RT-qPCR analysis of the 14 *AtrAP2/ERF* TFs and 14 predicted target genes was conducted to investigate their expression patterns under ethylene and 1-MCP treatments. The results showed that the expression of AP2 genes (*AP2/03/04/13/14*) was upregulated under both ethylene and 1-MCP treatments compared to the control, indicating that the different treatments did not have much effect on the expression of AP2 genes. Most of the ERF genes (*AtrERF001/005/009/020/043/054/068/082/090*) showed higher expression under ethylene treatment while, they were significantly down-regulated under 1-MCP treatment compared with the control fruit (**Figure 4A**). Moreover, predicted target genes (cell wall modification-related genes), including *AtrPGs*, *AtrPEs*, *AtrGALs*, and *AtrXTHs*, were significantly increased under ethylene treatment, but decreased under 1-MCP treatment (**Figure 4B**).

**Figure 5:**
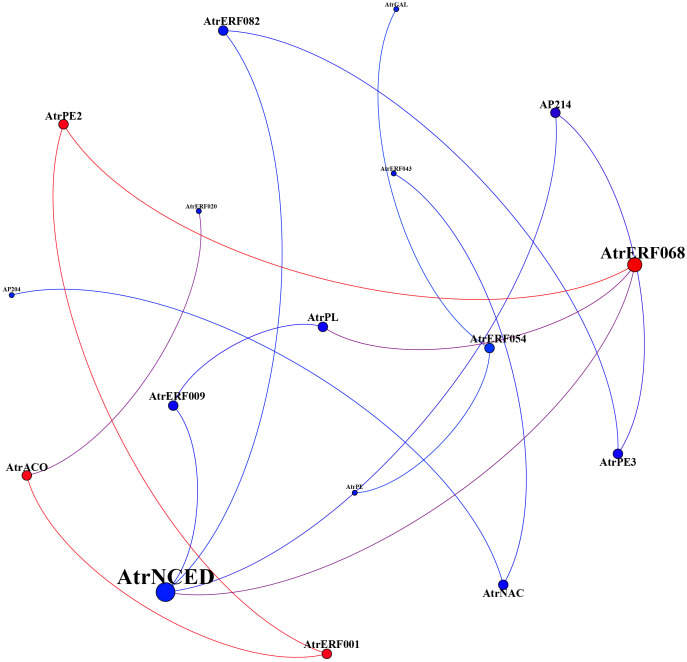
Weighted network correlation analysis of key *AtrERF* genes and cell wall-related genes.

The co-expression regulatory network was constructed by gene expression profiles under ethylene and 1-MCP stress to further elucidate the interaction between AP2/ERF transcription factor members and cell wall-related genes. *AtrERF068*, *AtrERF001*, *AtrPE2*, *AtrACO*, and *AtrNCED* were the most associated genes, which were concentrated in fruits under ethylene treatment (**Figure 5**). Among these *AtrERF* genes, one ERF gene (*AtrERF001*) showed higher expression levels in full maturity and demonstrated a strong association with the cell wall-related genes. Our future work will involve cloning and isolating the homologous gene for *AtrERF001* from *A. trifoliata* to confirm its role in fruit ripening.

### Transcription factor *AtrERF001* may indirectly regulate the expression of *AtrACO* and *AtrPE2* genes through yeast one-hybrid and EMSA experiments

**Figure 6:**
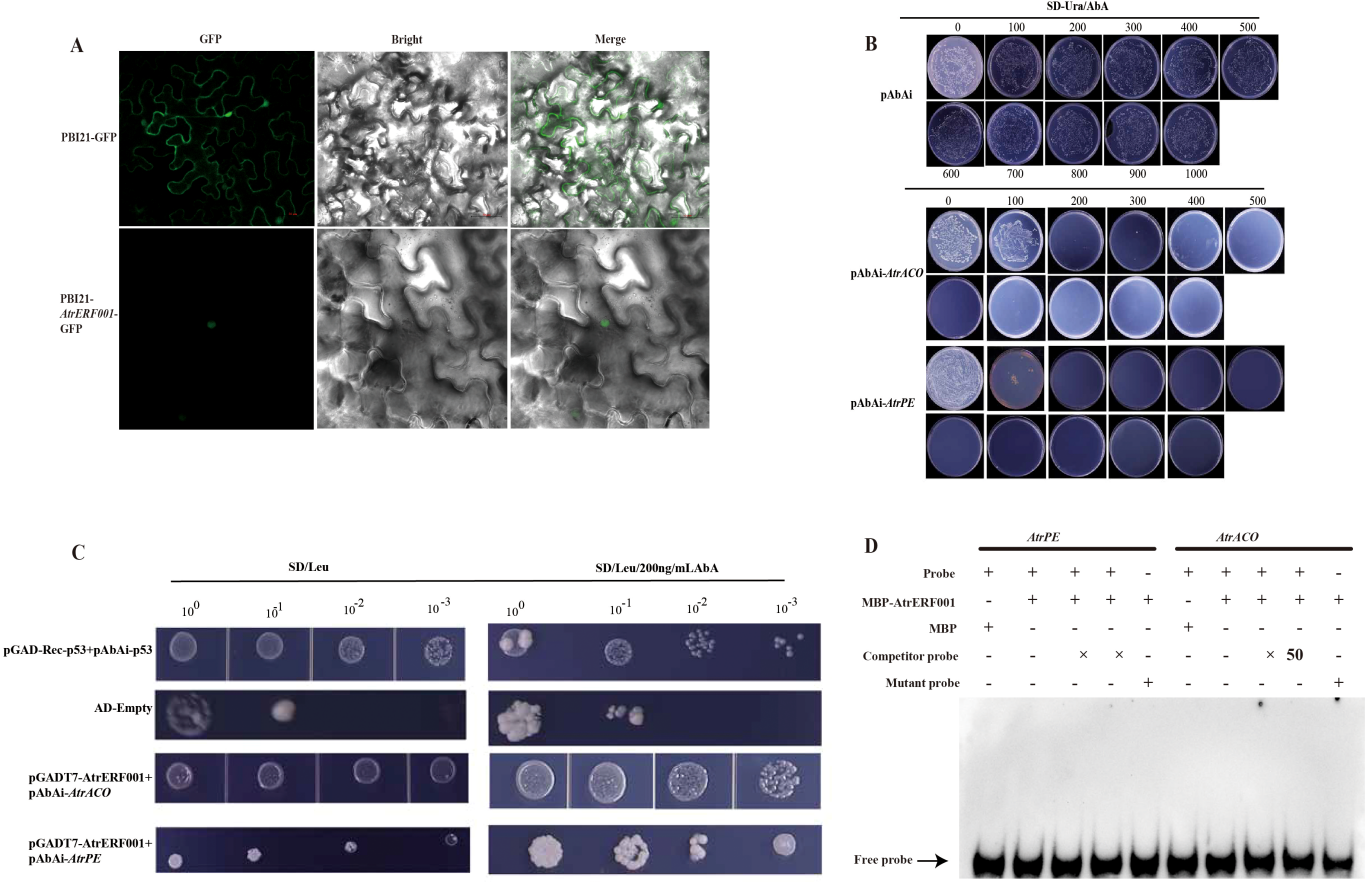
Analysis of subcellular localization, yeast one-hybrid and EMSA assays. **(A)** Subcellular localization of the *AtrERF001* gene. Red numbers indicate the scale. **(B)** Determination of the minimum inhibitory concentration of AbA on the decoy strain. **(C)** Yeast one-hybrid (Y1H) analysis showed that *AtrERF001* could bind to the promoters of *AtrACO* and *AtrPE*. **(D)** The EMSA assay of AtrERF001 protein using biotinylated double-stranded *AtrACO* and *AtrPE* probes. The purified MBP or recombinant MBP-AtrERF001 protein was mixed with the probes, and the protein–DNA complexes were separated on native polyacrylamide gels. + and – indicate the presence and absence of the indicated probe or protein, respectively; arrows indicate the positions of protein–DNA complexes or free probes.

In order to study the protein properties of ERF transcription factors in *A. trifoliata*, subcellular localization and yeast one-hybrid (Y1H) were conducted to elucidate the function of *AtrERF001 in vivo* and *in vitro*. The *AtrERF001* ORF sequence was cloned into the PBI121 vector without termination codons. The fluorescence signals from the *AtrERF001* fusion protein were found to be exclusively localized in the nucleus following the transient expression of these constructs in tobacco leaves, suggesting that *AtrERF001* is a nuclear protein (**Figure 6A**).

To investigate the transcriptional activity of *AtrERF001*, the Y1H assay was used to verify the binding of *AtrERF001* to the *AtrACO* and *AtrPE* promoters. Sequence analysis of the *AtrACO* and *AtrPE* promoters (2000 bp) revealed that there are *AtrERF* binding sites (GCCGAC). The CDS of *AtrERF001* was cloned into the pGADT7 vector for effector construction, and the promoter sequences of *AtrACO* and *AtrPE* were cloned to create the reporter construct. The minimum inhibitory concentration of aureobasidin A (AbA) was determined by self-activation assay (**Figure 6B**). The pGADT7-AtrERF001 and pAbAi-*AtrACO*/*AtrPE* co-transformants grew well on SD/-Leu/AbA200 medium (**Figure 6C**), while the pGADT7+p53-AbAi negative control could not grow normally on the same medium, indicating that AtrERF001 specifically binds to the binding sites of the *AtrACO and AtrPE* promoters. To confirm the interaction of AtrERF001 with the *AtrACO and AtrPE* promoters, the AtrERF001 protein were purified and used for EMSA. However, the site mutation assay result showed that AtrERF001 could not bind to the GCC box (GCCGAC elements) in the promoters of *AtrACO and AtrPE* (**Figure 6D**). Therefore, AtrERF001 may indirectly regulate *AtrACO and AtrPE* by interacting with other TFs.

### Transient overexpression and repressed expression of *AtrERF001* in *A. trifoliata* fruits

**Figure 7:**
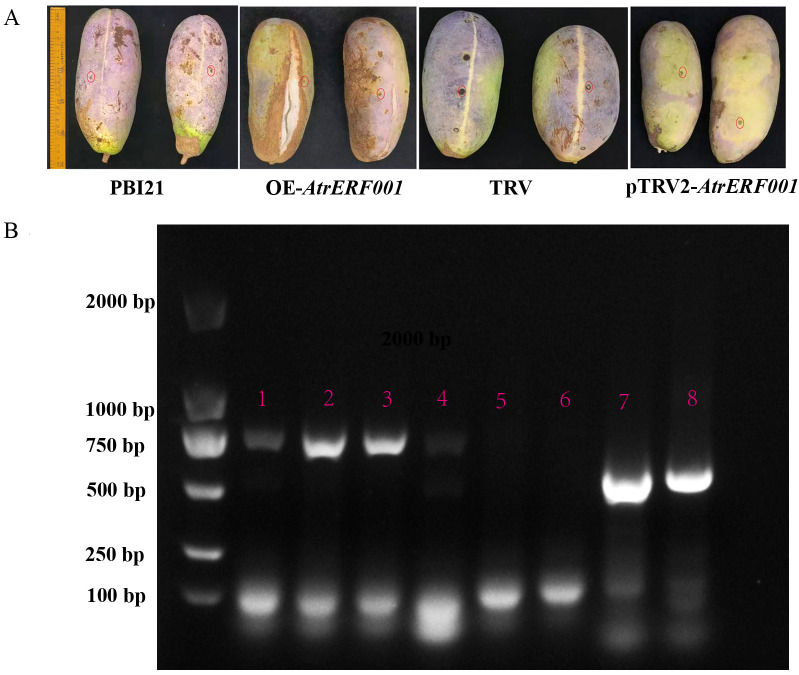
Phenotypic identification of overexpression and gene silencing of *AtrERF001* in *A. trifoliata* fruits. **(A)** Phenotypic observation of transient overexpression (OE-*AtrERF001*), inhibited expression (pTRV2-*AtrERF001*) and control (PBI121 and TRV) fruits of *A. trifoliata* after two weeks of infection. Red circles indicate the injection sites. **(B)** PCR and electrophoresis to detect the band size of genetically transformed fruits. The red numbers 1–4 were the results of overexpression (OE-*AtrERF001*) fruit detection, the red numbers 5 (PBI121) and 6 (TRV) were the results of control fruit, and the red numbers 7 and 8 were the results of inhibition (pTRV2-*AtrERF001*) fruit PCR and electrophoresis analysis.

**Figure 8:**
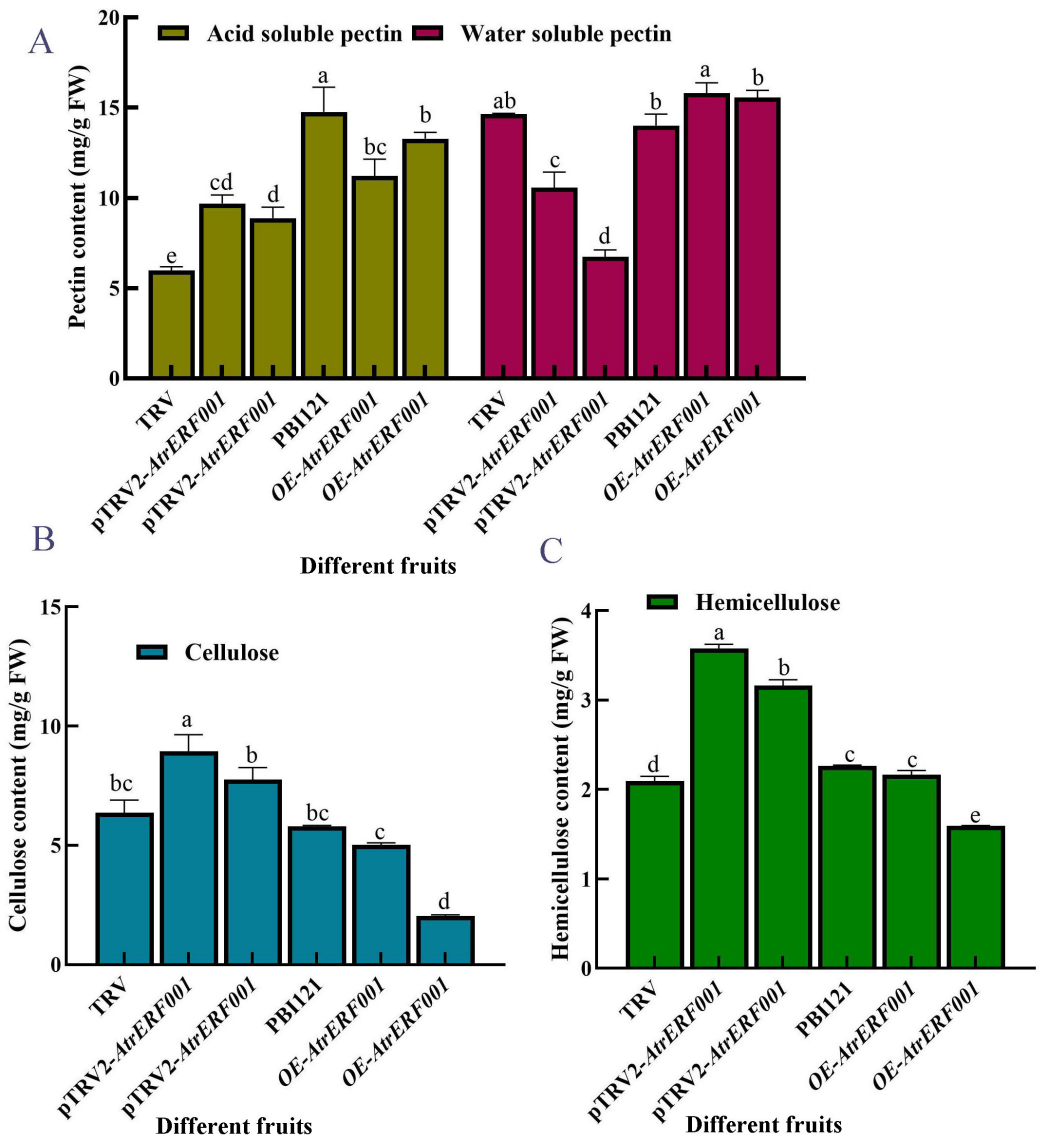
Determination of cell wall content in over-expressed and gene-silenced *A. trifoliata* fruits. **(A)** Pectin, **(B)** cellulose, and **(C)** Alphabet markers showed that there was a significant difference between overexpression (OE-AtrERF001) and empty vector control (PBI121) under the same treatment conditions. The error bar represents the standard error, and the lowercase letter represents P < 0.05, which has significant difference. The independent experiment was repeated 3 times.

To verify the regulation of *AtrERF001* on *AtrACO and AtrPE* expression in *A. trifoliata*, overexpression vector (PBI21-*AtrERF001*-GFP: OE-*AtrERF001*) and transient silencing vector (pTRV2-*AtrERF001*) were constructed. Then they were transiently transformed into immature *A. trifoliata* fruits, respectively (**Figure 7A**). Compared with the control fruits (PBI21-GFP and TRV vectors), the ventral suture line was more clearly visible in over-expression fruits (OE-*AtrERF001*), and fruit cracking began to occur after 14 days of infection. RNA was extracted from injected fruits (*AtrERF001* gene overexpression and transient silencing fruits), and the *AtrERF001* protein coding sequences carried by PBI121 and TRV vectors were amplified respectively to detect the results of transient transformation (primer sequence, **Supplementary Table S4**). It can be seen from **Figure 7B** that *AtrERF001* gene overexpression fruits (red No. 2-3) and silencing fruits (red No. 7-8) had obvious *AtrERF001* gene band size. The control fruits (empty vector, red number 5: PBI21-GFP; red No.6: TRV) did not detect the *AtrERF001* gene band, indicating that the target gene *AtrERF001* was accumulated in transiently transformed fruit. Moreover, compared with the control, the overexpressing *AtrERF001* (OE-*AtrERF001*) fruit showed higher WSP content, whereas the ASP, cellulose, and hemicellulose contents decreased. Silencing of *AtrERF001* in *A. trifoliata* significantly increased the ASP, cellulose, and hemicellulose contents (**Figure 8**). This suggests that the degradation of cell wall materials, specifically the conversion of carbonate-soluble pectin to WSP, marks the initiation of fruit ripening (Jiang Y.L. et al., 2022).

**Figure 9:**
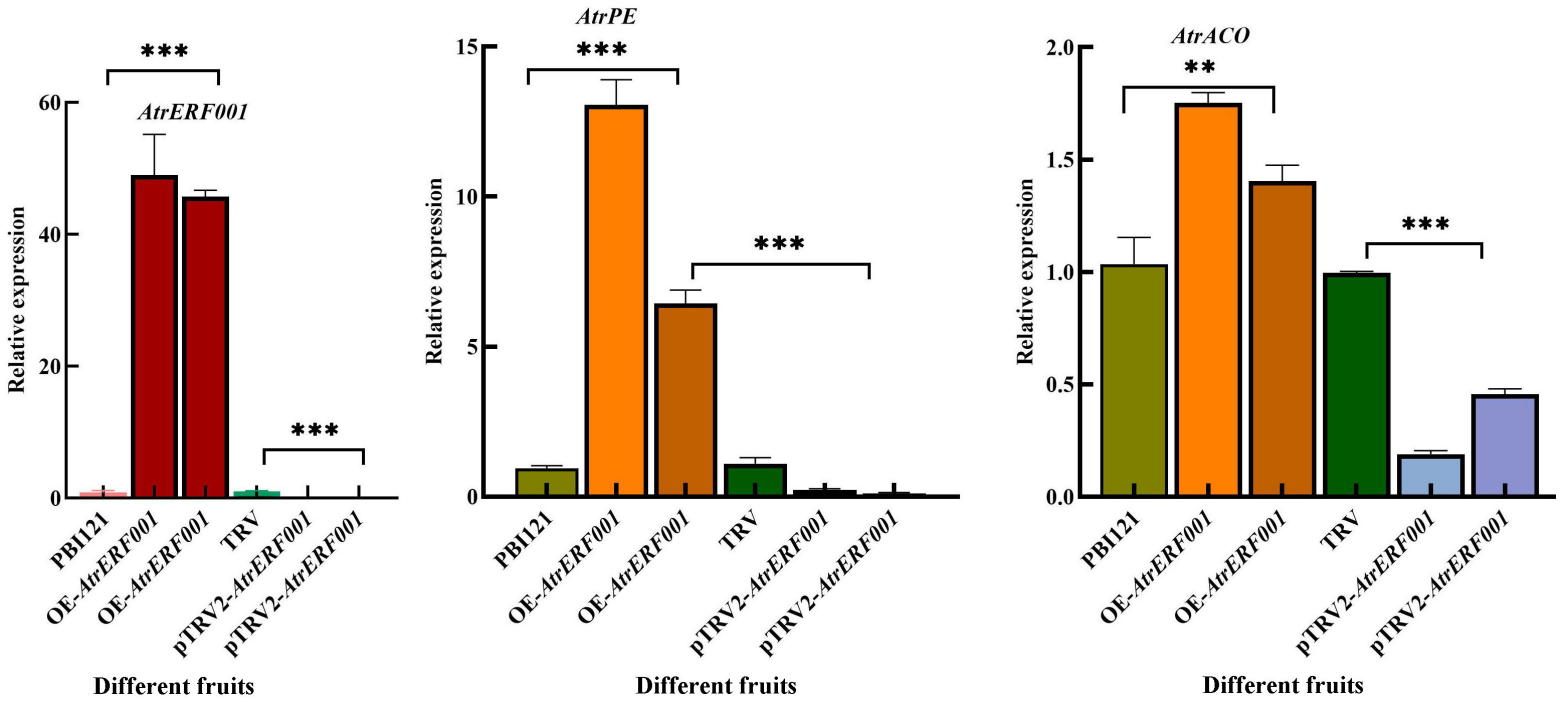
Gene expression analysis of hub genes including *AtrERF001*, *AtrPE*, and *AtrACO* examined using RT-qPCR in fruits with overexpression and gene silencing of *AtrERF001* in *A. trifoliata* fruits, **represents *p <*0.01, ***represents *p <*0.001.

RT-qPCR was further used to analyze the changes in gene expression in *A. trifoliata* fruits after overexpression and gene silencing of *AtrERF001*. The abundances of *AtrERF001* transcripts increased significantly after *AtrERF001* infiltration, and the levels of *AtrACO and AtrPE* transcription were also increased. Remarkably, the expression of the three genes increased approximately 30-fold, 5-fold, and 1-fold, respectively, in OE fruits than the control. Meanwhile, the expression of these three genes was significantly reduced in silenced fruits (**Figure 9**). These results indicate that *AtrERF001* may promote the maturation of *A. trifoliata* by indirectly affecting the expression of key genes *AtrACO and AtrPE* involved in ethylene metabolism and cell wall degradation.

### Identification and expression analysis of *AtrERF001* overexpression transgenic tomato

**Figure 10:**
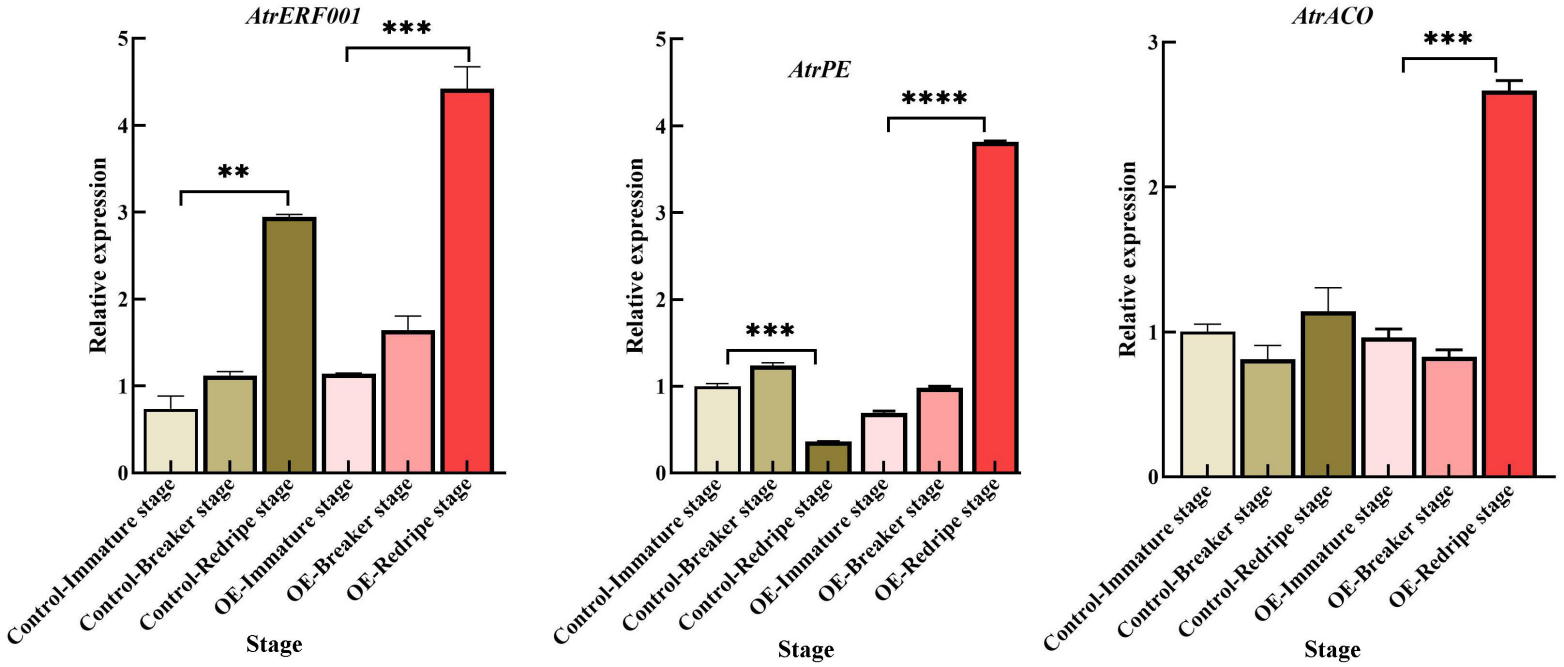
Gene expression analysis of hub genes including *AtrERF001*, *AtrPE*, and *AtrACO*, examined using RT-qPCR in fruits with overexpression and gene silencing of *AtrERF001* in tomato fruits, **represents *p <*0.01, ***represents *p <*0.001, ****represents *p <*0.0001.

Since the regeneration system and genetic transformation of *A. trifoliata* are more difficult, we chose tomato as the material for the next step of validation, which provides a good basis for the validation of *AtrERF001* genes. To confirm the success of the transgene, PCR analysis of T0 tomato leaves was performed using specific primers of *AtrERF001*. By sequencing, the PCR product of the transgenic tomato leaves was consistent with the *AtrERF001* gene at 753 bp, with 99% similarity and genetic distance (**Supplementary Figure S9**). Transgenic tomato fruits entered the breaking stage earlier than control fruits. RT-qPCR was then used to detect changes in the expression of *AtrERF001* at different developmental stages. During fruit development, the expression levels of *AtrERF001* increased dramatically, peaking at the red-ripe stage in both the control and transgenic fruits. The expression of *AtrERF001* in the OE red-ripe stage fruit increased approximately 1.5-fold compared to that in the control fruits. Moreover, the level of *AtrACO and AtrPE* transcription was increased significantly after *AtrERF001* infiltration at the red-ripe stage (**Figure 10**). These findings provide strong evidence for the crucial role played by *AtrERF001* in modulating fruit ripening.

## Discussion

AP2/ERF TFs bind to downstream target gene promoters through core elements of DRE (A/GCCGAC) or GCC cassette (AGCCGCC) to regulate the expression of downstream genes involved in plant growth, developmental stress response, fruit ripening, and softening (Liu and Zhang, 2017; Zhu et al., 2018; Kabir et al., 2021; Xing et al., 2021). Thus, studying the AP2/ERF TFs is useful for understanding their roles in *A. trifoliata* fruit ripening. A total of 131 *AP2/ERF* genes in *A. trifoliata* were identified, slightly more than in jujube (Zhang and Li, 2018), longan (Chen et al., 2018), citrus (Xie et al., 2014), and pomegranate (Wan et al., 2022). Notably, in *A. trifoliata*, no *AP2/ERF* genes belonging to the Vb-L subgroup were found. The difference in *AP2/ERF* genes may be due to the evolution and duplication of the genes, which is consistent with the classification of *ERFs* in grapevines (Zhu et al., 2018).

Whole-genome duplication, especially tandem and segmental duplication events, and the entire genome are important evolutionary and expansion drivers for gene families (Jiang P.F. et al., 2022). In the case of *A. trifoliata*, 55 and 3 paralogous gene pairs were identified to have undergone SD and TD, respectively, suggesting that SD promotes the expansion of gene families, such as the *SlMTP* and *MAPK* gene families (El- Sappah et al., 2021; Li et al., 2022). Ka/Ks ratios smaller than 1 were observed in most duplicated *AtrERF* gene pairs, suggesting that purifying selection pressure predominated during the development of *A. trifoliata ERF* genes, most likely due to environmental changes (Morgan et al., 2010).

Analyses of gene expression profiles can be used to make preliminary predictions of gene functions. Gene expression analyses pointed out fourteen ripening-related ERF genes in *A. trifoliata*. In the current study, the expression of *AtrERF001* was significantly increased by ethylene and inhibited by 1-MCP and their transcriptional patterns were similar to those of *AtrPE* and *AtrACO.* Based on these results, we speculate that *AtrERF001* may bind to the promoter of *AtrERF001* downstream gene and regulate its expression, thereby mediating cell wall degradation and promoting the maturation of *Akebia trifoliata.* Previous studies indicated that ERF TFs can be involved in fruit ripening by regulating downstream target genes. During fruit ripening, *MdERF* binds to the DRE motif in the promoter of apple *MdACS1* (ACC synthase gene) and regulates ethylene production. *PbERF24* participates in pear ripening by directly regulating *PbACO54* expression (Hao et al., 2018). The expression level of *DkPL1* and *DkPE1* are considerably increased by transient overexpression of *DkERF8* and *DkERF18* in persimmon fruit, indicating that *DkERFs* are crucial for the ripening process (He et al., 2020). In this study, by constructing a co-expression regulatory network analysis, *AtrERF001* was identified to be co-expressed with *AtrPE* and *AtrACO*, and sequence analysis showed that both genes contained ERF binding sites in their promoter regions. Furthermore, subcellular localization analysis showed that *AtrERF001* is a nuclear protein, and Y1H assay demonstrated that *AtrERF001* can specifically bind to the *AtrPE* and *AtrACO* promoters and activate *AtrPE* and *AtrACO* transcription. However, through EMSA experiments, it was found that there was no interaction between *AtrERF001* and *AtrPE* and *AtrACO*, suggesting that *AtrERF001* may work together with unknown transcription factors to activate *AtrPE* and *AtrACO* expression. It may be necessary to further verify the interaction between these gene regulatory networks through Ch IP-Seq and other techniques (Wang L. et al., 2020).

Transient overexpression of *DkERF8* and *DkERF18* can encourage the conversion of ASP to WSP, which is associated with the softening of persimmon fruit (He et al., 2020). As fruit ripeness increased, banana fruit hardness decreased rapidly, which was associated with an increase in WSP content and a decrease in ASP content (Duan et al., 2008). Moreover, transient overexpression of *AtrERF001* enhanced the conversion of ASP to WSP in *trifoliata* fruits, which is a characteristic of *A. trifoliata* fruit ripening (Jiang Y. et al., 2022). Lower cellulose and hemicellulose contents were observed in OE fruits; however, they increased after silencing *AtrERF001*, suggesting that *A. trifoliata* fruit ripening is due to the degradation of pectin, cellulose, and hemicellulose (Wang et al., 2019b). The abundances of *AtrERF001* transcripts increased significantly after *AtrERF001* infiltration, and the levels of *AtrACO and AtrPE* transcription were also increased in both OE-*A. trifoliata* and tomato fruits, while decreased in the gene silenced fruits. The gene expression levels of *AtrACO and AtrPE* in overexpression *A. trifoliata* and tomato fruits of *AtrERF001* were higher than those in the control during fruit ripening (**Figures 9**, **10**), but the site mutation assay result showed that *AtrERF001* could not bind to the GCC box in the promoters of *AtrACO and AtrPE*. The reason may be that yeast one-hybrid is the environment *in vivo* (within yeast cells), and there may be other cofactors or post-translational modifications, while EMSA is an *in vitro* experiment, which may lack these conditions, resulting in binding undetectable. Therefore, *AtrERF001* may indirectly regulate *AtrACO and AtrPE* by interacting with other TFs. Follow-up studies will be undertaken to research the role of other transcription factor families or protein-protein interactions in *A. trifoliata* fruit ripening (He et al., 2020).

In summary, the *AP2/ERF* gene family from *A. trifoliata* was firstly identified and analyzed, including taxonomy, evolution and synthesis, expression profile, and hormone treatment. Moreover, based on pre-transcriptomic studies, ethylene and 1-MCP treatments, Y1H and genetic transformation analysis, a candidate AP2/ERF gene-*AtrERF001* was verified to be involved in fruit ripening by indirectly affecting expression of the *AtrACO* and *AtrPE*, which is consistent with pear fruit ripening (Hao et al., 2018). This study improves our understanding of the ERF family in *A. trifoliata* comprehensively and provides a theoretical basis for the transcriptional regulation mechanism of *A. trifoliata* fruit ripening.

## Data Availability

The datasets presented in this study can be found in online repositories. The names of the repository/repositories and accession number(s) can be found in the article/**Supplementary Material**.

## Supplementary material

Supplementary Figure 1 Tomato leaf tissue culture and shoot growth.

Supplementary Figure 2 Schematic representation of the chromosomal distribution of *AtrAP2/ERF* genes.

Supplementary Figure 3 Multiple sequence alignments of the AP2 domains of *AtrAP2/ERF*.

Supplementary Figure 4 Phylogenetic tree representing relationships among AP2 domains of *A. trifoliata* using MEGA-X software. (A) Phylogenetic tree representing relationships among AP2 domains of *A. trifoliata*, *Arabidopsis thaliana*, and *Oryza sativa*. (B) Phylogenetic tree representing relationships among AP2 domains of *A. trifoliata*.

Supplementary Figure 5 Phylogenetic relationships, conserved motifs, and gene structure of *AP2/ERF* genes from *A. trifoliata*. (A) Neighbor-joining tree based on the full-length protein sequences of 131 A*. trifoliata* AP2/ERFs. (B) Motif composition of *A. trifoliata* AP2/ERF proteins. (C) Intron-exon structures of *A. trifoliata* AP2/ERFs genes.

Supplementary Figure 6 Gene Ontology (GO) and Kyoto Encyclopedia of Genes and Genomes (KEGG) enrichment analysis of *AtrAP2/ERF* potential target genes in *A. trifoliata*. (A) GO analysis of *AP2/ERF* target genes in *A. trifoliata*. Categories of cellular components, molecular functions, and biological processes were defined by GO classification. (B) KEGG enrichment analysis of *AtrAP2/ERF* potential target genes in *A. trifoliata.*

Supplementary Figure 7 AtrAP2/ERF protein interaction network based on Arabidopsis homologs. (A) Interaction network of the AP2/ERF genes of *A. trifoliata*. (B) Interaction network between the AP2/ERF genes and cell wall-related genes in *A. trifoliata*.

Supplementary Figure 8 Hierarchical clustering of *A. trifoliata* AP2/ERF gene expression profiles in nine samples, including different tissues and developmental stages, based on Log2(FPKM + 1) values.

Supplementary Figure 9 Identification of transgenic tomato plants. (A) Phenotypic identification of transgenic plants (T-26) and control fruits. (B) Sequence comparison between the sequencing results of T-26 and the *AtrERF001* gene. (C) Phylogenetic relationships of T-26, *AtrERF001*, and *SlERFs*.

Supplementary Table 1 FPKM values in nine samples, including different tissues and developmental stages.

Supplementary Table 2 Sequences of specific primers used for RT-qPCR experiments.

Supplementary Table 3 Primer sequences used in the Y1H assays.

Supplementary Table 4 cDNA and expression vector primer sequences of *AtrERF001*.

Supplementary Table 5 List of gene IDs and characteristics of the 131 *AtrAP2/ERF* genes.

Supplementary Table 6 Duplicated gene pairs of tandemly and segmental duplication events.

Supplementary Table 7 Ka/Ks ratios of the tandemly and segmental duplicated gene pairs in *A. trifoliata*.

Supplementary Table 8
*AtrAP2/ERF* genes showing a syntenic relationship with dicotyledonous and monocotyledonous.

Supplementary Table 9 Number of cis-elements in the promoter region of *AtrAP2/ERF* genes.

Supplementary Table 10 Gene ID of *AtrAP2/ERF* potential target genes in *A. trifoliata*.
