# Supplementary figures and images for: Genome-wide analysis of AP2/ERF family in Akebia trifoliata and characterization of an AtrERF001 gene regulate fruit ripening
Source: Front Plant Sci. 2025 Aug 26;16:1607254. doi: 10.3389/fpls.2025.1607254 (PMC12417410; doi:10.3389/fpls.2025.1607254)

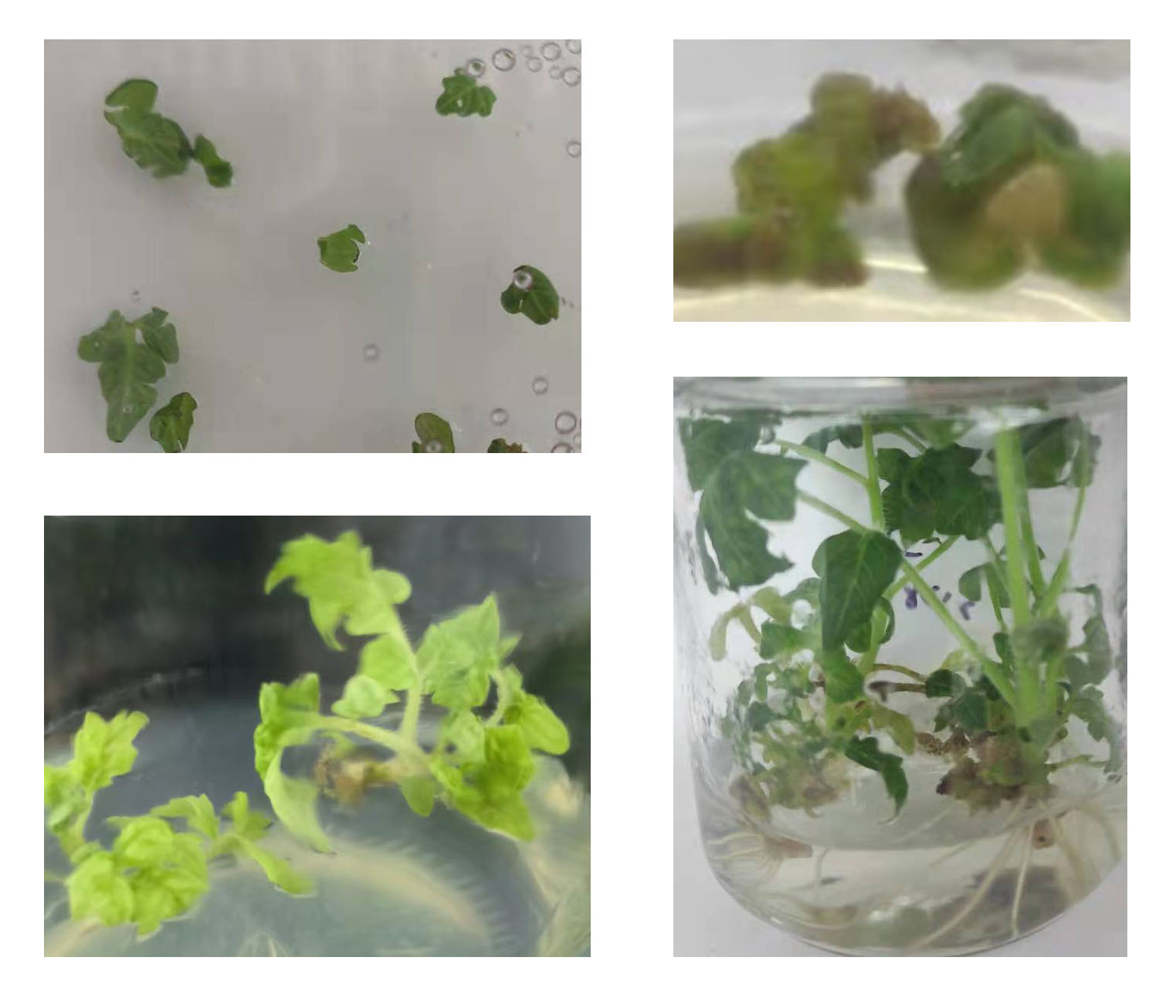

Supplement: Supplementary Figure 1 — Tomato leaf tissue culture and shoot growth. [file Image1.jpg]

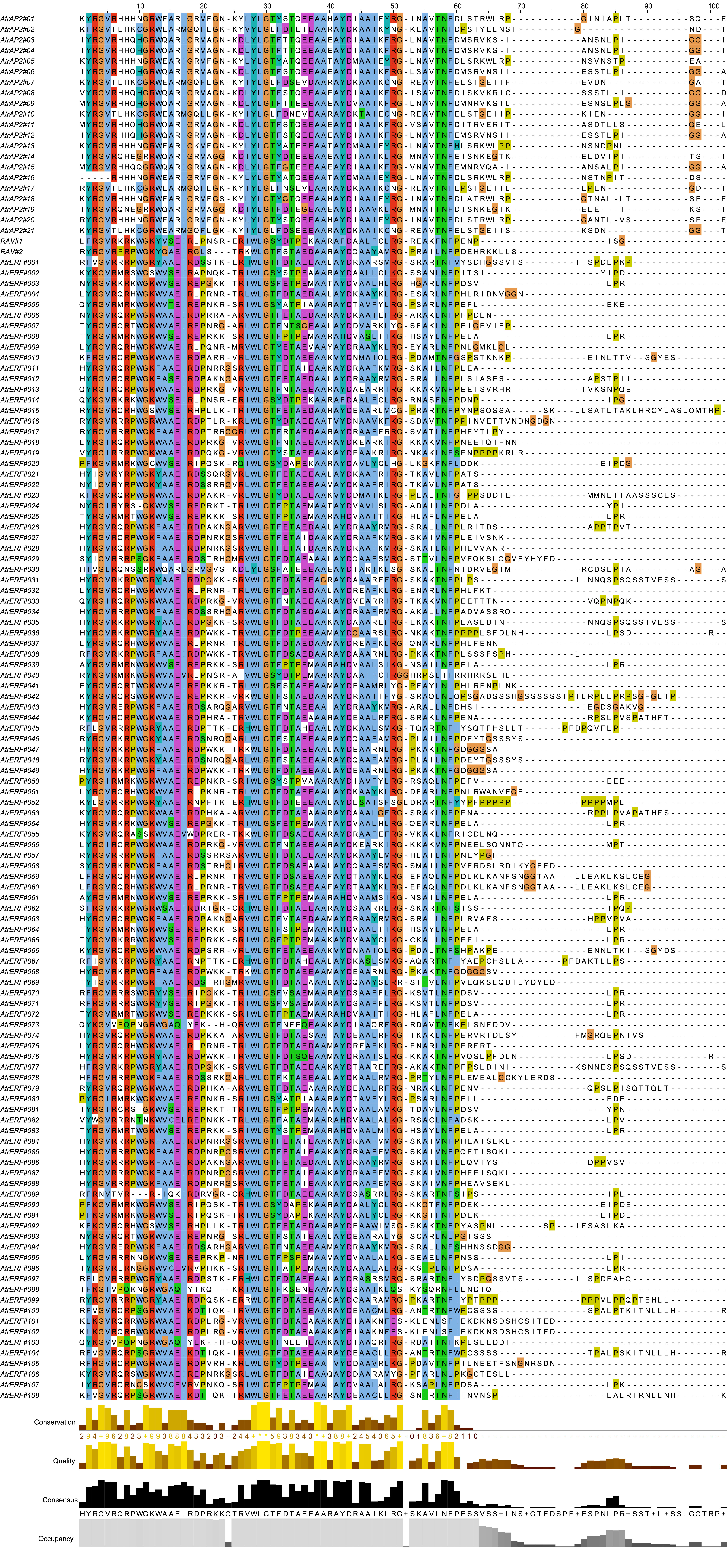

Supplement: Supplementary Figure 3 — Multiple sequence alignments of the AP2 domains of AtrAP2/ERF. [file Image3.jpg]

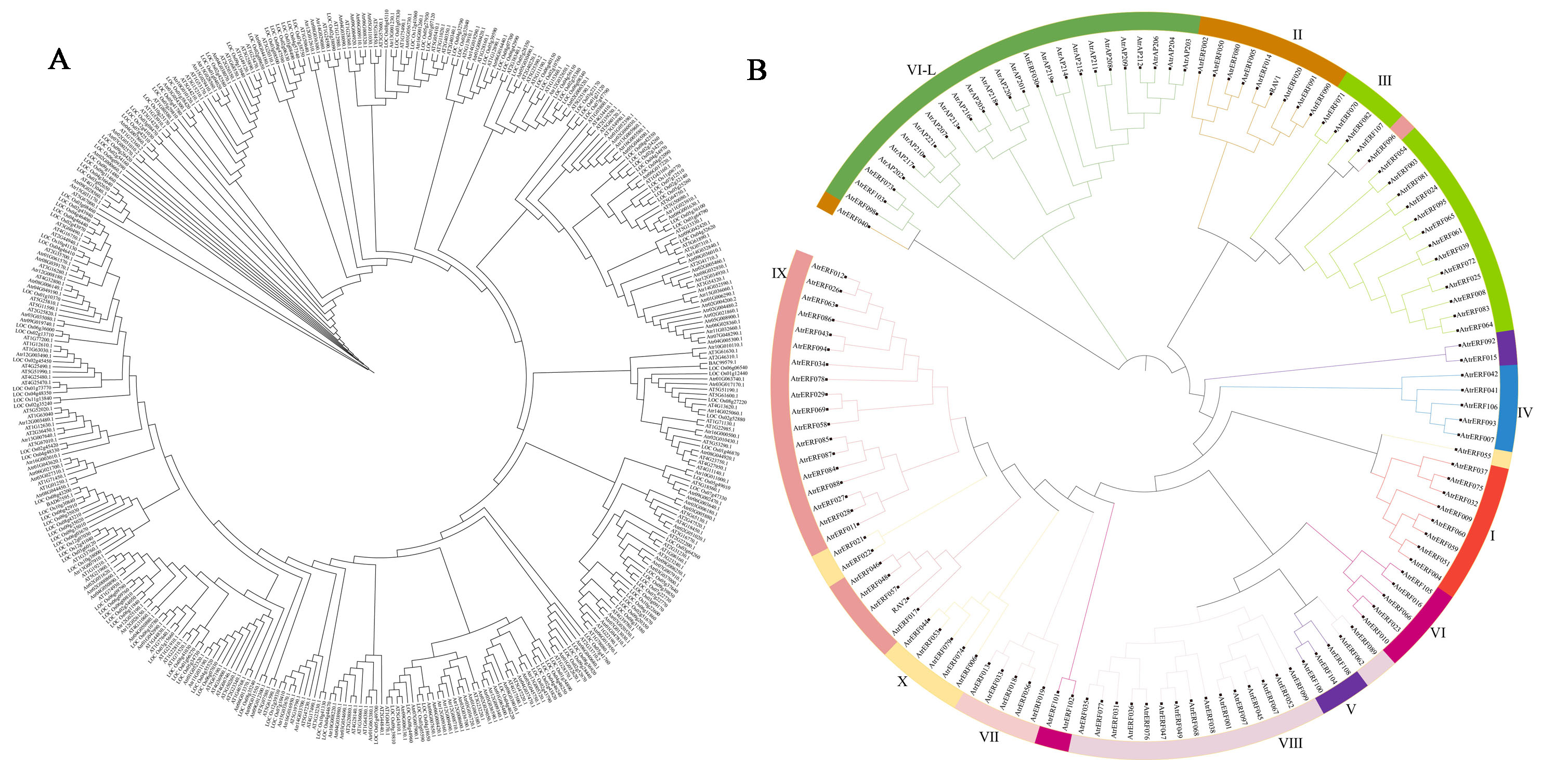

Supplement: Supplementary Figure 4 — Phylogenetic tree representing relationships among AP2 domains of A. trifoliata using MEGA-X software. (A) Phylogenetic tree representing relationships among AP2 domains of A. trifoliata, Arabidopsis thaliana, and Oryza sativa. (B) Phylogenetic tree representing relationships among AP2 domains of A. trifoliata. [file Image4.jpg]

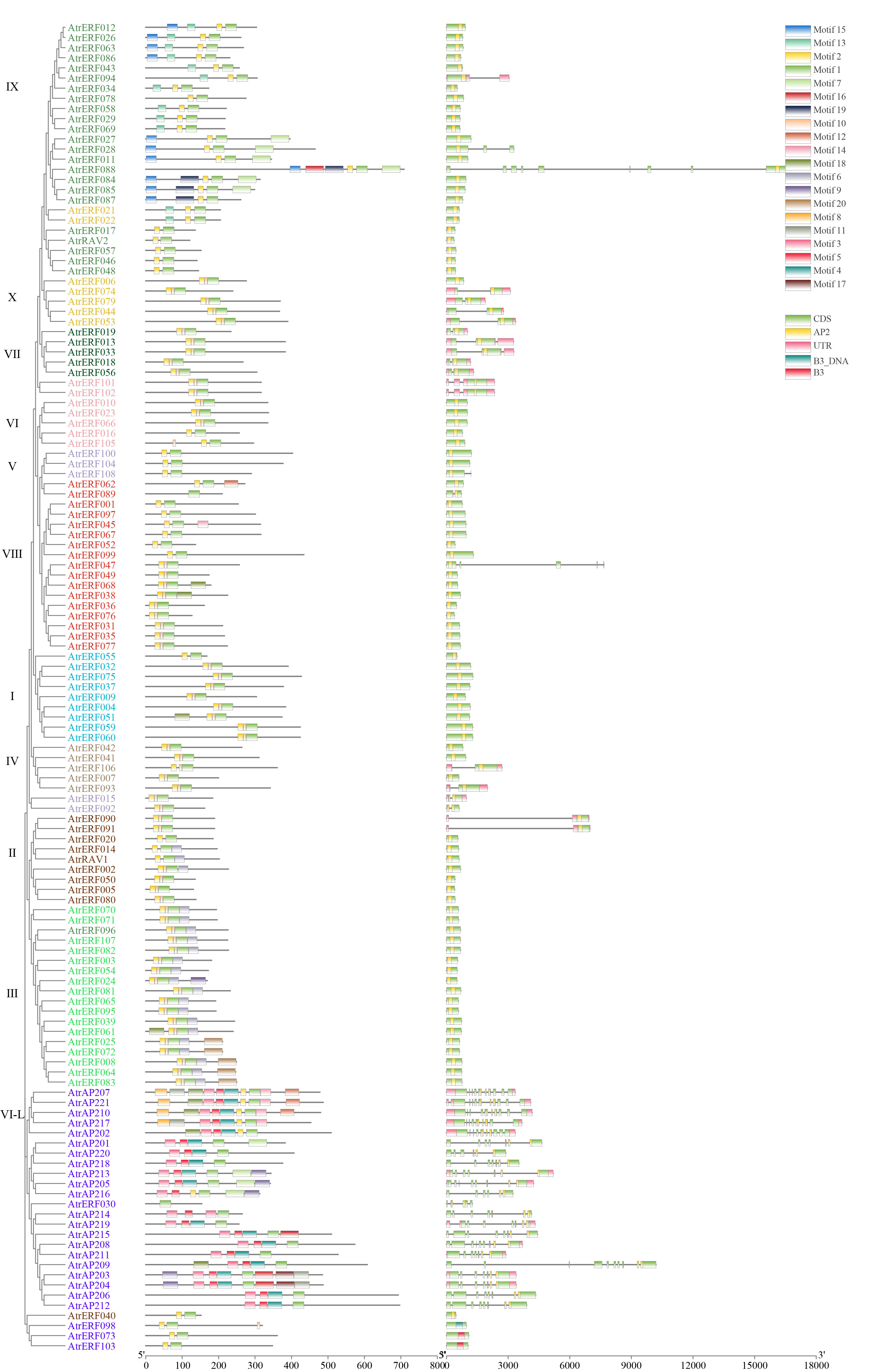

Supplement: Supplementary Figure 5 — Phylogenetic relationships, conserved motifs, and gene structure of AP2/ERF genes from A. trifoliata. (A) Neighbor-joining tree based on the full-length protein sequences of 131 A. trifoliata AP2/ERFs. (B) Motif composition of A. trifoliata AP2/ERF proteins. (C) Intron-exon structures of A. trifoliata AP2/ERFs genes. [file Image5.jpg]

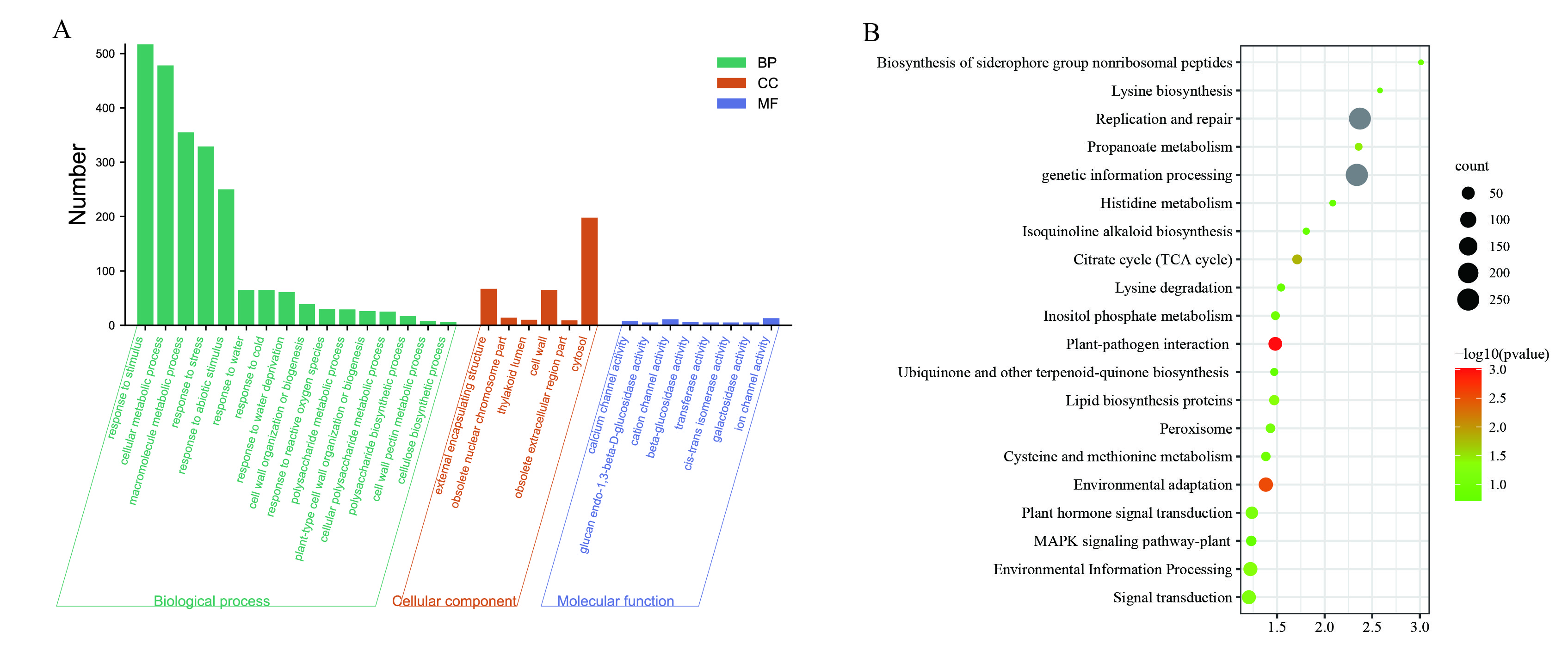

Supplement: Supplementary Figure 6 — Gene Ontology (GO) and Kyoto Encyclopedia of Genes and Genomes (KEGG) enrichment analysis of AtrAP2/ERF potential target genes in A. trifoliata. (A) GO analysis of AP2/ERF target genes in A. trifoliata. Categories of cellular components, molecular functions, and biological processes were defined by GO classification. (B) KEGG enrichment analysis of AtrAP2/ERF potential target genes in A. trifoliata. [file Image6.jpg]

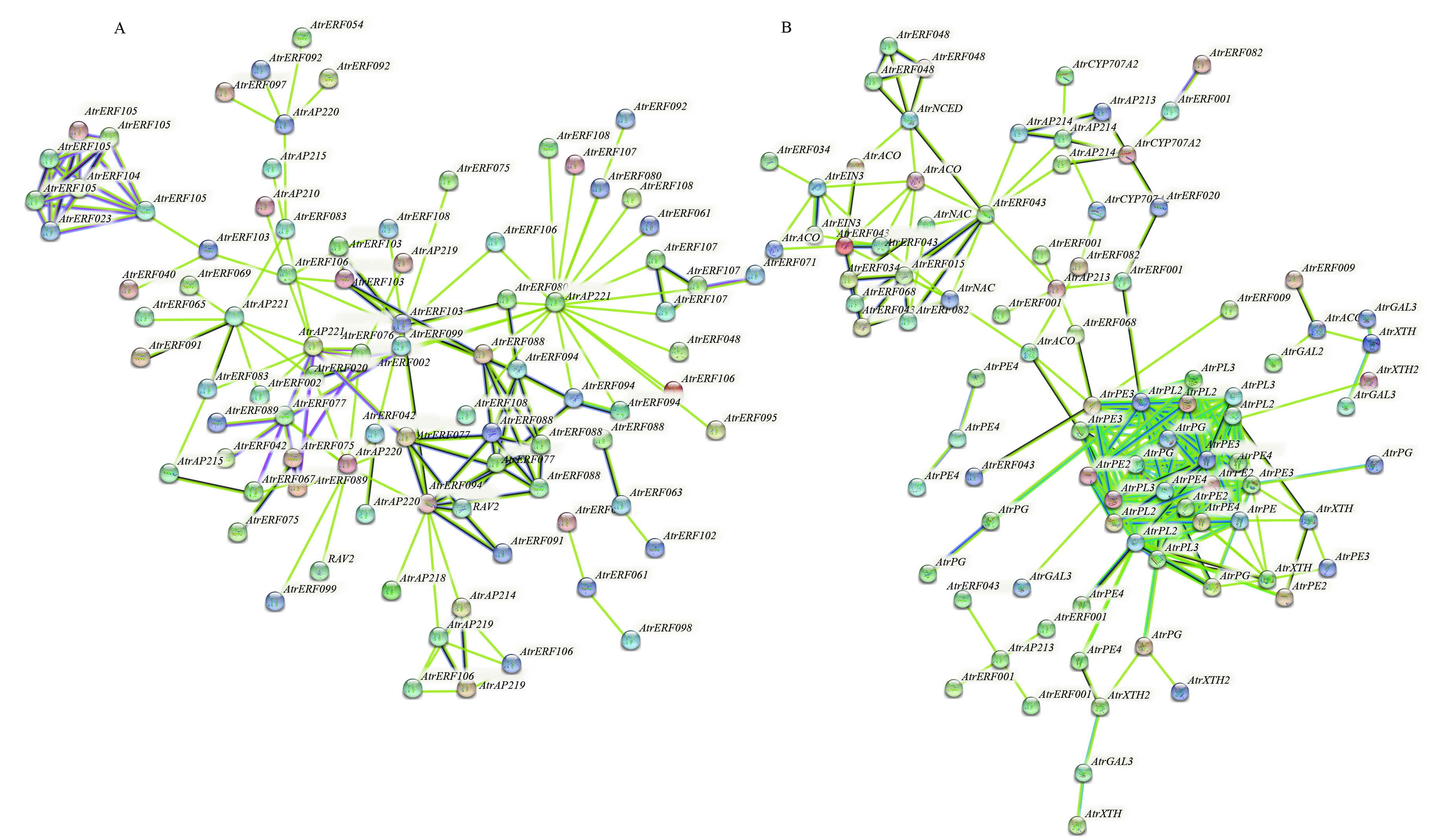

Supplement: Supplementary Figure 7 — AtrAP2/ERF protein interaction network based on Arabidopsis homologs. (A) Interaction network of the AP2/ERF genes of A. trifoliata. (B) Interaction network between the AP2/ERF genes and cell wall-related genes in A. trifoliata. [file Image7.jpg]

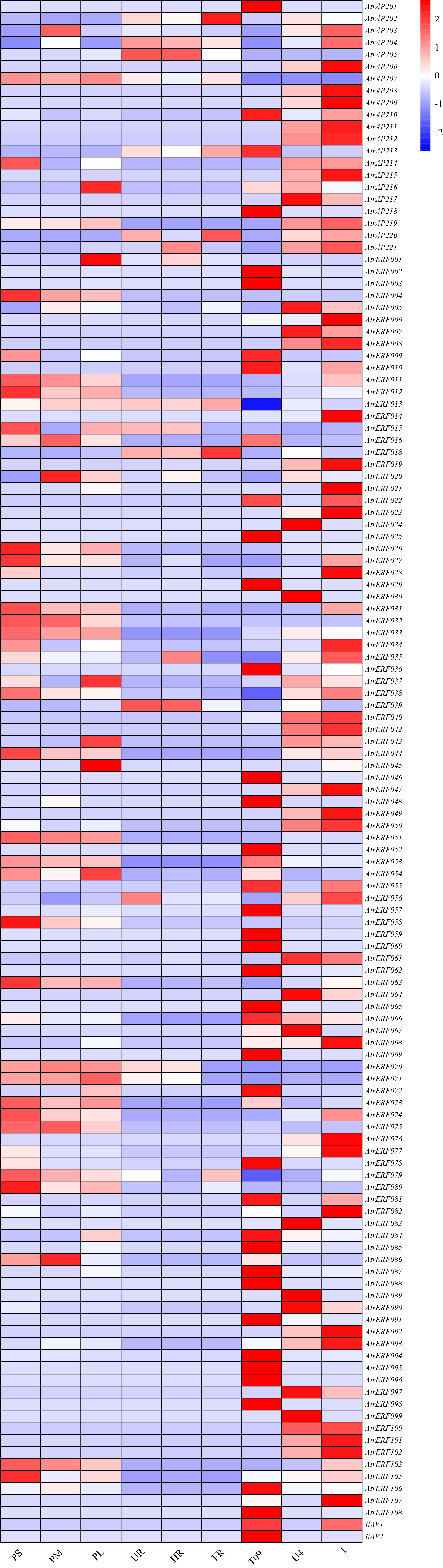

Supplement: Supplementary Figure 8 — Hierarchical clustering of A. trifoliata AP2/ERF gene expression profiles in nine samples, including different tissues and developmental stages, based on Log2(FPKM + 1) values. [file Image8.jpg]

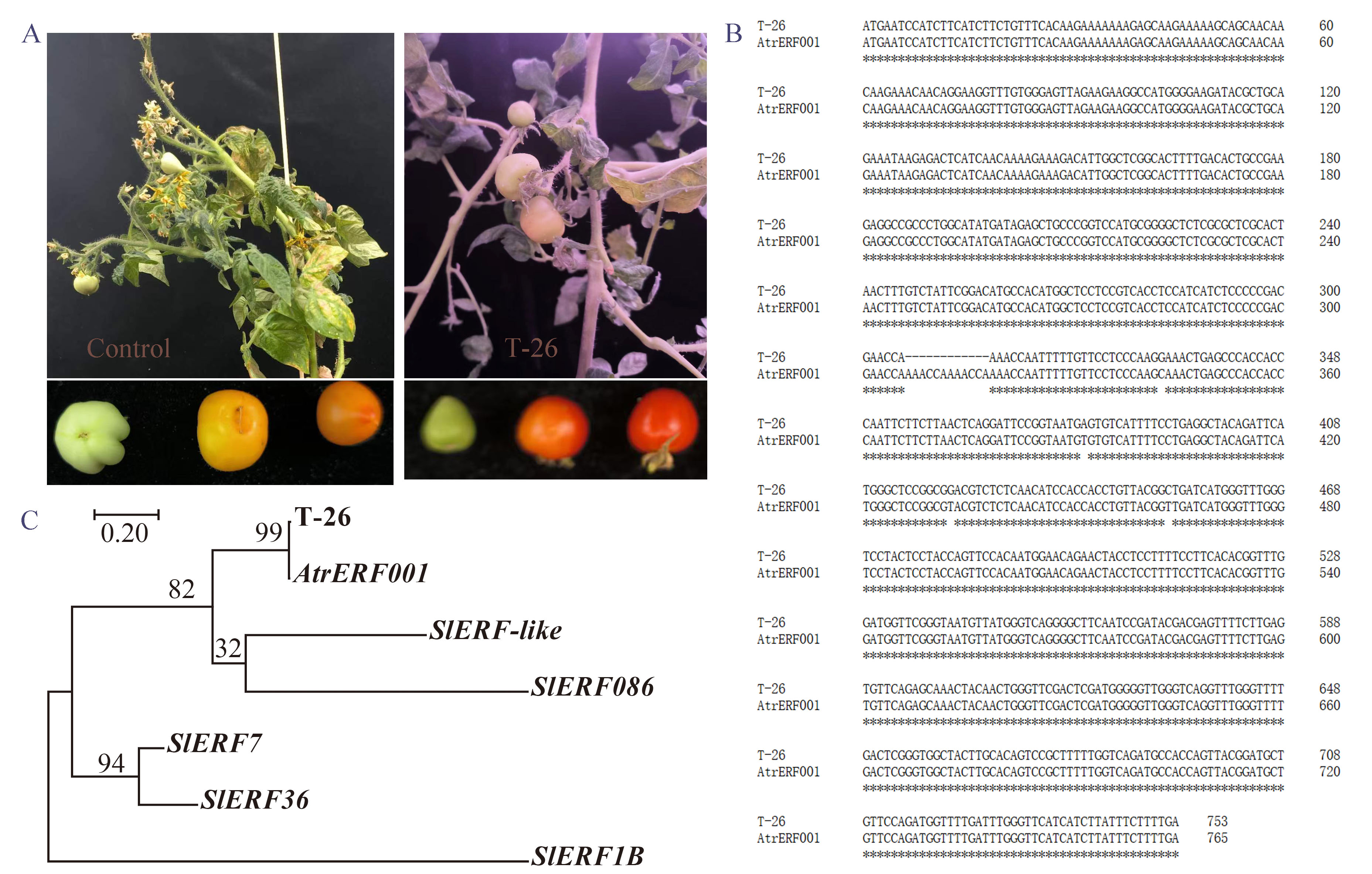

Supplement: Supplementary Figure 9 — Identification of transgenic tomato plants. (A) Phenotypic identification of transgenic plants (T-26) and control fruits. (B) Sequence comparison between the sequencing results of T-26 and the AtrERF001 gene. (C) Phylogenetic relationships of T-26, AtrERF001, and SlERFs. [file Image9.jpg]
